# Genetic vulnerabilities upon inhibition of DNA damage response

**DOI:** 10.1093/nar/gkab643

**Published:** 2021-07-28

**Authors:** Chao Wang, Mengfan Tang, Zhen Chen, Litong Nie, Siting Li, Yun Xiong, Klaudia Anna Szymonowicz, Jeong-Min Park, Huimin Zhang, Xu Feng, Min Huang, Dan Su, Traver Hart, Junjie Chen

**Affiliations:** Departments of Experimental Radiation Oncology, The University of Texas MD Anderson Cancer Center, Houston, TX 77030, USA; Departments of Experimental Radiation Oncology, The University of Texas MD Anderson Cancer Center, Houston, TX 77030, USA; Departments of Experimental Radiation Oncology, The University of Texas MD Anderson Cancer Center, Houston, TX 77030, USA; Departments of Experimental Radiation Oncology, The University of Texas MD Anderson Cancer Center, Houston, TX 77030, USA; Departments of Experimental Radiation Oncology, The University of Texas MD Anderson Cancer Center, Houston, TX 77030, USA; Departments of Experimental Radiation Oncology, The University of Texas MD Anderson Cancer Center, Houston, TX 77030, USA; Departments of Experimental Radiation Oncology, The University of Texas MD Anderson Cancer Center, Houston, TX 77030, USA; Departments of Experimental Radiation Oncology, The University of Texas MD Anderson Cancer Center, Houston, TX 77030, USA; Departments of Experimental Radiation Oncology, The University of Texas MD Anderson Cancer Center, Houston, TX 77030, USA; Departments of Experimental Radiation Oncology, The University of Texas MD Anderson Cancer Center, Houston, TX 77030, USA; Departments of Experimental Radiation Oncology, The University of Texas MD Anderson Cancer Center, Houston, TX 77030, USA; Departments of Experimental Radiation Oncology, The University of Texas MD Anderson Cancer Center, Houston, TX 77030, USA; Bioinformatics and Computational Biology, The University of Texas MD Anderson Cancer Center, Houston, TX 77030, USA; Departments of Experimental Radiation Oncology, The University of Texas MD Anderson Cancer Center, Houston, TX 77030, USA

## Abstract

Because of essential roles of DNA damage response (DDR) in the maintenance of genomic integrity, cellular homeostasis, and tumor suppression, targeting DDR has become a promising therapeutic strategy for cancer treatment. However, the benefits of cancer therapy targeting DDR are limited mainly due to the lack of predictive biomarkers. To address this challenge, we performed CRISPR screens to search for genetic vulnerabilities that affect cells’ response to DDR inhibition. By undertaking CRISPR screens with inhibitors targeting key DDR mediators, i.e. ATR, ATM, DNAPK and CHK1, we obtained a global and unbiased view of genetic interactions with DDR inhibition. Specifically, we identified *YWHAE* loss as a key determinant of sensitivity to CHK1 inhibition. We showed that *KLHL15* loss protects cells from DNA damage induced by ATM inhibition. Moreover, we validated that *APEX1* loss sensitizes cells to DNAPK inhibition. Additionally, we compared the synergistic effects of combining different DDR inhibitors and found that an ATM inhibitor plus a PARP inhibitor induced dramatic levels of cell death, probably through promoting apoptosis. Our results enhance the understanding of DDR pathways and will facilitate the use of DDR-targeting agents in cancer therapy.

## INTRODUCTION

Normal cells often encounter a variety of endogenous and exogenous DNA lesions (1,2). To protect themselves from these various types of DNA damage, cells evolve a complex network named DNA damage response (DDR) to coordinate DNA damage sensing, signal transduction, and repair of these DNA lesions. DDR also regulate cell cycle progression and control many other cellular processes (3–5). Dysfunctional DDR leads to unrepaired or mis-repaired genome that causes genomic instability and eventually human diseases such as cancers (6). Thus, although genomic instability is a hallmark of cancer, it may also provide targetable vulnerabilities for cancer therapy (7,8).

Although traditional DNA damage-based therapies such as radiotherapy and chemotherapy have shown great benefits in clinic, these therapies kill both cancer cells and normal cells and therefore have dose-limiting toxicities (9,10). In contrast, targeted cancer therapies based on the unique genetic contexts of specific cancers may reduce toxicity and enhance anti-tumor efficacy (11). Among these therapies, those targeting DDR are promising for several reasons. First, DDR is essential for ensuring genomic stability, which is often dysregulated in cancers (3,12–14). Second, mechanisms underlying DDR pathways are well studied, and highly specific inhibitors targeting DDR pathways have already been developed for preclinical and clinical applications (15). More importantly, therapies targeting DDR pathways, such as PARP inhibitors, have proven to be effective for the treatment of breast or ovarian cancers with *BRCA1/2* mutations (16,17). However, the current utility of other DDR inhibitors is limited because the specific genetic vulnerabilities associated with these inhibitors are unknown.

The critical regulators of DDR are protein kinases, e.g. phosphatidylinositol-3-kinase-like kinase [PI3KK] family kinases (5). These protein kinases include ATM (ataxia-telangiectasia mutated), ATR (ATM- and Rad3-related), and DNAPK (DNA-dependent protein kinase) (7,11). In response to various types of DNA damage, these kinases activate and phosphorylate hundreds of downstream proteins to regulate a variety of cellular processes, including DNA repair, cell cycle progression, and apoptosis (5). ATM is a master kinase that regulates two major pathways involved in DNA double-strand break repair, i.e. homologous recombination (HR) and nonhomologous end joining (NHEJ) pathways (18). Loss of ATM protein kinase activity results in hypersensitivity to ionizing radiation (IR) and predisposes individuals to cancers (19–21). ATR protein kinase is another master DDR kinase that functions in cellular response to a broad range of DNA lesions, including replication stresses, ultraviolet light (UV), IR and environmental mutagens (22–26). Moreover, ATR protein kinase also plays important roles in telomere maintenance and cell cycle checkpoint control (27). For its role in cell cycle regulation, ATR protein kinase activates a key downstream kinase, checkpoint kinase 1 (CHK1), and initiates a wave of phosphorylation events that are important for cell cycle progression (28–32). The versatile functions of ATM and ATR protein kinases make them the master transducers of DNA damage signals, which are essential for genome maintenance. Compared to ATM and ATR protein kinases, DNAPK regulates a smaller number of targets and plays a role primarily in NHEJ pathway (33,34). These three PI3KK kinases play critical and sometimes redundant roles in DDR. Their functions are distinguishable from other PI3KK family members such as mammalian target of rapamycin (mTOR) and SMG1 (suppressor of morphogenesis in genitalia-1) (35,36). It is also noteworthy that, although they have different biological functions, all these PI3KKs have structural similarities within their PIKK domains, which makes it challenging to develop specific inhibitors targeting the individual kinases (37).

Knowledge of these key DDR kinases has been accumulated mostly from studies of their substrates and/or their interaction partners (38–41). These previous studies revealed the detailed mechanisms and key functions of these kinases. However, in-depth genetic interactions with these protein kinases have not yet been explored due to technical limitations. Nowadays, CRISPR-based genetic screens enable genome-scale analyses of gene-gene and gene-drug interactions in human cells, which can provide comprehensive views of protein functions and their genetic interactions (42). Indeed, recent CRISPR screens using RPE1 cells revealed a genetic network of cellular responses to a variety of genotoxic agents, demonstrating the power of CRISPR-based screens in functional exploration (42). Recent reports using CRISPR screens have also illustrated new vulnerabilities to specific DDR inhibitors, such as ATR inhibitor (ATRi), ATM inhibitor (ATMi), poly adenosine diphosphate ribose polymerase (PARP) inhibitor (PARPi), and CHK1 inhibitor (CHK1i) (43–46). However, these studies are not suitable to infer the difference among distinct DDR inhibitors because their screens were carried out in isolation using different cell lines and/or different CRISPR libraries. Accordingly, we conducted this study to obtain a comprehensive view of genetic interactions in response to DDR inhibitors. To accomplish this, we conducted genome-scale CRISPR screens in a single cell line with several PI3KK family kinase inhibitors (ATRi, ATMi, DNAPK inhibitor [DNAPKi], mTOR inhibitor [mTORi]) and related DDR inhibitors (CHK1i and PARPi). Our results elucidate the key differences among these specific DDR inhibitors, identify new synthetic lethal or survival relationships, and offer potential biomarkers for the clinical application of these inhibitors.

## MATERIALS AND METHODS

### Cell lines and cell cultures

HEK293A and HCT116 cells were obtained from ATCC. Dulbecco's modified Eagle's medium with 10% fetal calf serum was used to culture the HEK293A cells. Mcoy5A medium with 10% fetal calf serum was used to culture the HCT116 cells. All the cells passed the test of mycoplasma.

### CRISPR screens

The screens were conducted as described in a previous paper (43). Briefly, the Toronto KnockOut library was transfected into HEK293A cells with the packaging vector psPAX2, the envelope vector pMD2.G, and X-treme Gene transfection reagent (Roche). The virus-containing media were collected 24 h after transfection, centrifuged at 1500 rpm for 5 mins, and frozen for subsequent sgRNA screens. For sgRNA screening, 120 million HEK293A or HCT116 cells were infected with lentiviruses encoding the Toronto KnockOut library at a low multiplicity of infection ratio (< 0.3). Twenty-four hours after infection, the medium was replaced with fresh medium containing puromycin (2 μg/ml). After selection, cells were split into different groups containing ∼20 million cells each, passaged every 3 days, and maintained at 200-fold coverage. At day 0 and every 3 days from day 6 to day 21, 25 million cells (>300-fold coverage) were collected for genomic DNA extraction. Genomic DNA was extracted from cell pellets using the QIAamp Blood Maxi Kit (Qiagen), precipitated using ethanol and sodium chloride, and resuspended in Buffer EB (10 mM Tris–HCl, pH 7.5). Polymerase chain reaction was used to amplify gRNA inserts; primers harboring Illumina TruSeq adapters with i5 and i7 barcodes were used, and the resulting libraries were sequenced on an Illumina HiSeq 2500 system. The BAGEL algorithm was used to calculate essentiality scores. DrugZ analysis was used to calculate the difference between the dimethyl sulfoxide- and drug-treated groups.

### Generation of KO cells

KO cells were generated using pLentiCRISPRv2. Cells were transiently transfected with the indicated plasmids and selected using puromycin (2 μg/ml). Single cells were then plated into 96-well plates. After 10 days, clones were picked and checked with the indicated antibodies using western blotting.

### Antibodies

The antibodies used in this study are as following: γH2A.x (Millipore, Cat. 05-636); CHK1 (Cell Signaling, Cat. 2360); pCHK1(s345) (Cell Signaling, Cat. 2348); CHK2 (Cell Signaling, Cat. 6334); pCHK2(T68) (Cell Signaling, Cat. 2197); pRPA2(s33) (Bethyl, Cat. A300-246A); RPA2 (Cell Signaling, Cat. 2208); 53BP1 (Santa Cruz Biotechnology, Cat#sc-515841); BRCA1 (Santa Cruz Biotechnology, Cat#sc-6954); pDNA PKcs (S2056) (Abcam, Cat#ab18192); beta-tubulin (Sigma-Aldrich, Cat#T5168); CtIP (Cell Signaling, Cat.9201).

### Inhibitors

The inhibitors used in this study are as following: AZD6738 (Selleck Chemicals, S7693), LY2603618 (Selleck Chemicals, S2626), CVT313 (Selleck Chemicals, S6531), AZD0156 (Selleck Chemicals, S8375), AZD1390 (Selleck Chemicals, S8680), NU7441 (BioVision, B1875-5), Rapamycin (Selleck Chemicals, S1039), Olarparib (Selleck Chemicals, S1060).

### sgRNAs, shRNA and siRNAs

The sgRNAs, shRNAs and siRNAs used in this study are as following: YWHAE sgRNA1 (AAGCGAATAGGATGCGTTGG); YWHAE sgRNA2 (ACTTCAGACATGCAGGGTGA); YWHAE sgRNA3 (CCTAAGCGAATAGGATGCGT); KLHL15 sgRNA1 (AAGAAGGGACCATAGAACAA); KLHL15 sgRNA2 (ACAACCCAGAGACTGATCAG); ATM sgRNA (GATGGCAGATATCTGTCACC); RAP80 sgRNA (GGAGGTGAACAGCCAGGAGG); CHD1L sgRNA (AGTTGGAGACCACCTGACTG); APEX1 sgRNA (GTAACGGGAATGCCGAAGCG); XRCC1 sgRNA (GGTACAGCTTACCTGGGACG); PNKP sgRNA (ACCAGGGCTTGCCCGTCCGA); PARP1 sgRNA (GCAGAAAGTCAAGAAGACAG); PARP2 sgRNA (AGGTTCGGAGCTCAATATCG); CtIP siRNA (Qiagen, SI03211859); BRCA1 siRNA (Qiagen, SI02664368); ATM shRNA1 (Horizon, RHS4430-200187878); ATM shRNA2 (Horizon, RHS4430-200190335)

### Western blotting

Cells were lysed in NETN buffer (20 mM Tris [pH 7.6], 1 mM ethylenediaminetetraacetic acid, 1% NP40, 150 mM NaCl) supplemented with protease-inhibitor cocktail tablets (Roche). Proteins were separated by sodium dodecyl sulfate polyacrylamide gel electrophoresis, transferred to membranes, and immunoblotted with antibodies as indicated in the figures.

### Crystal violet viability assays

For crystal violet viability assays, cells were seeded (200 cells per well in 6-well plate or 1500 cells per well in 12-well plate), exposed to chemicals/drugs as indicated, and cultured for 14 days or 7 days as indicated. Cells were stained with crystal violet (Sigma). Colonies were counted manually. All cell-survival assays were performed at least in triplicate.

### Flow cytometry

Cells were fixed in 70% ethanol and permeabilized with phosphate-buffered saline (PBS) with 0.5% Triton X-100. Cells were then incubated with primary antibodies diluted in PBS with 0.05% Triton X-100 (PBST) and 3% bovine serum albumin (PBST-BSA) for 1 h at room temperature. After three washes with PBS, fluorescently labeled secondary antibodies in PBST–BSA were added for 1 h. For FACS, propidium iodide staining was used to measure DNA content. Data were collected with a BD C6 flow cytometer (Becton Dickinson) and analyzed with Flowjo (Becton Dickinson).

### Immunofluorescence staining analysis

Cells were grown on coverslips for 24 h before treatment. After the indicated treatment, cells were fixed in 4% paraformaldehyde and permeabilized with PBS with 0.5% Triton X-100. Then, cells were incubated with primary antibodies diluted in PBST-BSA for 1 h at room temperature. After three washes with PBS, fluorescently labeled secondary antibodies in PBST–BSA were added for 1 h. Cells were then washed in PBS with Hoechst stain (1:10 000). Slides were imaged at 40× magnification on a Leica microscope.

### DNA fiber assay

Cells were labeled with 30 μM CIdU for 20 min, washed quickly twice with PBS and exposed to 250 μM IdU for another 20 min. Cells were harvested and resuspended in PBS. Cells were then lysed with lysis buffer (200 mM Tris–HCl pH7.4, 50 mM EDTA, 0.5% SDS), and DNA fibers stretched onto glass slides. The fibers were then denatured with 2.5 M HCl for 1 h, washed with PBS and blocked with 2% BSA in PBST for 30 min. The fibers were stained with anti-BrdU antibodies recognizing CIdU (Rat anti-BrdU, AbD Serotec OBT0030) and IdU (Mouse anti-BrdU, BD 347,580). Slides were imaged at 40× on a Leica microscope. The sum of IdU and CldU length were calculated to measure the DNA fiber length.

### HR reporter assay

U2OS cells stably expressing HR reporter DR‐GFP were gifts from Albert C. Koong lab in MD Anderson Cancer Center. 1 × 10^6^ U2OS DR‐GFP cells were transfected with the indicated sgRNAs or siRNAs. Twenty four hours later, 2 μg of pCBASce plasmid (an I‐SceI expression vector) were transfected into the cells with Lipo2000. Cells were cultured for another 48 hrs and subjected to flow cytometry analysis to determine percentages of GFP‐positive cells, which result from HR repair induced by DNA DSBs. Means were obtained from 3 independent experiments.

### Neutral comet assay

After trypsinization, cells were resuspended in PBS (Gibco) at a concentration of 2 × 10^5^ cells/ml. Seventy-five microliters of cell suspension were mixed in 500 μl LMAgarose (Trevigen), placed on gel bon films, covered with a 22-mm cover slide (VWR International), and left in the dark for 15 min at 4°C. After removal of the coverslip, cells were lysed in the dark for 1 h in Trevigen lysis at 4°C. Following washing with TBE (90 mM Tris-borate (pH 8.3) and 2 mM EDTA), the samples were subjected to electrophoresis at 35 V for 7 min in TBE. Afterwards, cells were fixed in 70% ethanol and dried at room temperature. The following day, nuclei were stained with SYBR green I (Invitrogen) in 10 mM Tris–HCl, pH 7.5, 1 mM EDTA, pH 8.0. Slides were imaged at 40× magnification on a Leica microscope. Relative tail moments were measured using the CometScore software (TriTek). For each group, tail moments of at least 200 cells were measured.

### Proteomic profiling with tandem mass tag (TMT)

HEK-293A (three biological replicates) and three different KLHL15 knock out clones were collected when the cell density reached 80–90% confluence. The cell pellets were washed with ice-cold PBS, then lysed in chilled lysis buffer (8.0 M urea in 0.1 M NH4HCO3, supplemented with 1 × protease inhibitor cocktail) with incubation on ice for 30 min and sonication at 4°C (2-min cycles of 5 s on and 10 s off at 30% output power for a tip-probe sonicator). After centrifugation, the supernatant was transferred to new chilled tubes and the protein concentration was determined by BCA protein assay following the manufacturer's instructions. The supernatant was diluted to equal protein concentrations. Proteins were reduced with 5 mM dithiothreitol at 56°C for 30 min, alkylated with 15 mM iodoacetamide at ambient temperature in the dark for 30 min, and then quenched by 15 mM cysteine. Samples were sequentially digested by Lys-C (enzyme: proteins, 1:100) for 4 h and then trypsin (enzyme: proteins, 1:50) overnight at 37°C. Before trypsin digestion, the protein solution was diluted with 4-fold 0.1 M NH_4_HCO_3_ (pH 8.0). The digestion was quenched by trifluoroacetic acid to a final 0.1% concentration, desaulted with the Sep-Pak SPE column (Waters, Milford, MA) and then dried with a SpeedVac.

The 6-plex TMT labeling was performed with previously reported protocol (69). Briefly, 50 μg peptides were dissolved in 25 μl of 50 mM HEPES (pH 8.5), then labeled with the TMT reagent for 1 h at room temperature and 1000 rpm. The reaction was quenched with hydroxylamine to a final concentration of 0.5% (v/v) for 15 min at room temperature and 1000 rpm. The equal TMT-labeling peptide solutions were pooled, then subjected toSep-PakC18 desalting and dried with a SpeedVac. The pooled TMT-labeling peptides were separated by high-pH reverse-phase HPLC with a Waters XBridge C18 column (3.7-μm particles, 4.6 × 250 mm) (70,71). Eluents were collected every 1 min in a 90-min gradient from 2% to 95% of buffer B (10 mM ammonium formate, 80% ACN) at a flow rate of 0.7 ml/min. The eluents were pooled into 16 fractions by previously reported method (71,72).

The fractions were dissolved with solvent A (0.1% formic acid in H2O), then analyzed on a Q Exactive HF-X mass spectrometer (Thermo Fisher Scientific, Waltham, MA) with a 65 min gradient from 5% to 50% solvent B (0.1% formic acid in 80% ACN). With data-dependent mode, the precursor ions were scanned with 375–1500 *m*/*z* and a resolution of 70 000 at *m*/*z* 200. The automatic gain control target was 1e6 with 100 ms of maximum injection time. The MS/MS fragmentation of 40 most intense ions above 1.5e4 were performed with higher collision dissociation with normalized collision energy of 28%, with 1 *m*/*z* isolation windows and 60 s of dynamic exclusion time. Ion fragments were detected in the Orbitrap at a resolution of 17 500 at *m*/*z* 200 with an automatic gain control 1e6 and 100 ms of maximum injection time. Precursor ions with one charge or five or more charges were excluded for fragmentation.

The acquired MS/MS raw data were searched against human proteomes database from uniprot (29 July 2020, updated, 93 798 sequences) by MaxQuant software (version 1.6.7.0) with a reversed decoy database by Andromeda search engine (73). The default parameters for TMT labeling were applied (73). The different expression analysis was performed with Perseus (version 1.6.7.0) with criteria, a permutation-based False Discovery Rate (FDR) <0.001 and S0 = 0.1 (74).

### Statistics

Statistical analyses were performed using GraphPad Prism software (version 8.0). All of the statistical methods used are described in the main text. Each experiment was repeated three times or more. Differences between groups were analyzed using the Student's *t*-test, unless otherwise noted. A *P* value <0.05 was considered statistically significant.

### Data availability

All relevant data not presented in the main figures or in the supplementary data are available from the authors.

## RESULTS

### Whole-genome CRISPR screens with DDR inhibitors in HEK293A Cells

**Figure 1.**
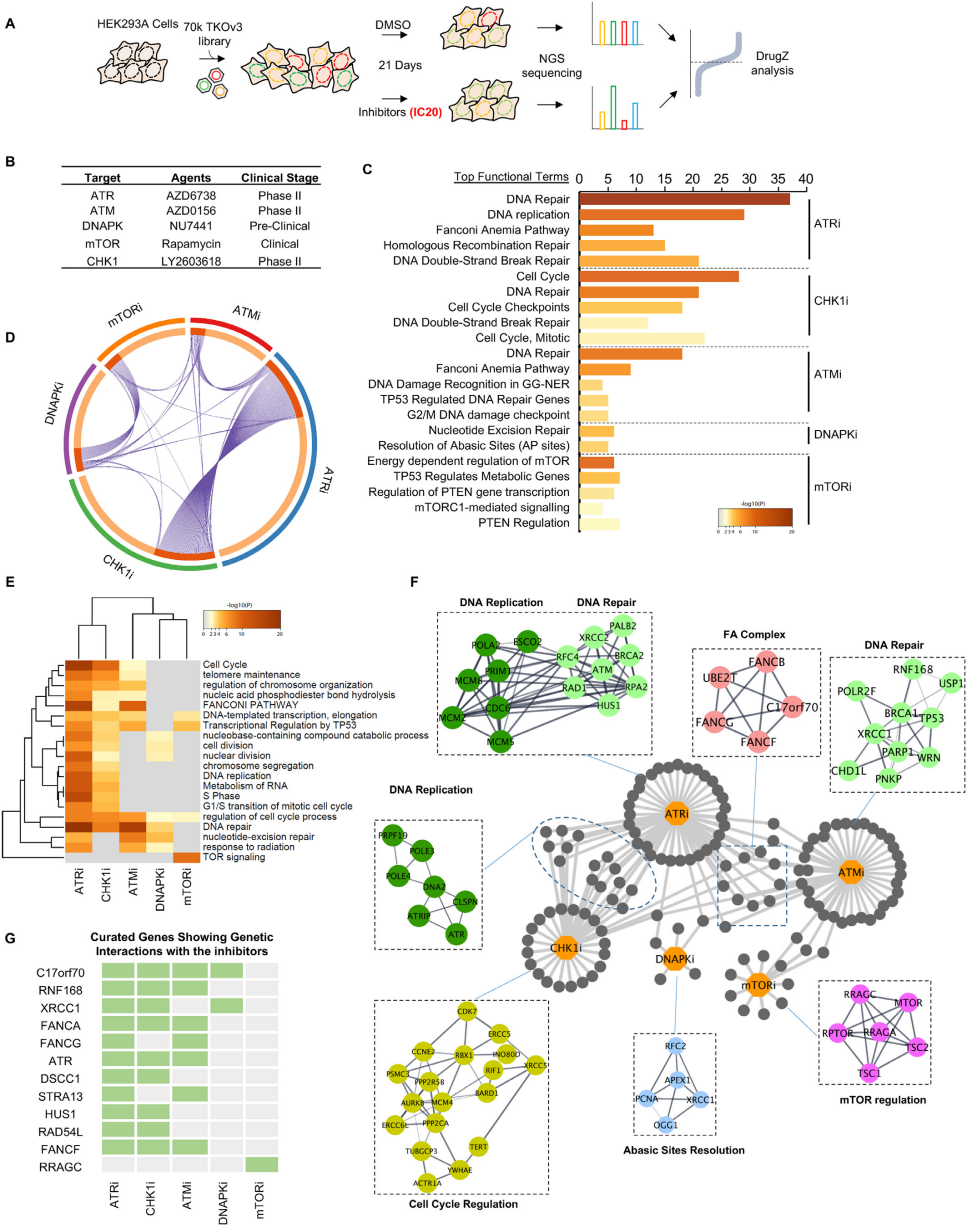
Genome-Wide CRISPR Screens with DDR Inhibitors in HEK293A Cells. (**A**) Schematic representation of the workflow for CRISPR screens performed in HEK293A cells. (**B**) Inhibitors used in the screens and their status in clinical trials. (**C**) Functional term analysis of the synthetic lethality genes identified in these CRISPR screens. The top five functional terms (REACTOME) enriched for each inhibitor are listed here. The numbers represent the number of genes enriched for each functional term. Different colors represent *P* values (Benjamini-Hochberg false discovery rate corrections). The functional term analyses were conducted with Metascape (https://metascape.org). (**D**) Circos graph of the synthetic lethal/sick genes for each inhibitor. The genes shared by different inhibitors are marked with lines. (**E**) Heatmap representation of the five CRISPR screens with different inhibitors. The top functional terms for each group were listed and the distances between the different groups were calculated by Metascape (http://metascape.org). Gray coloring means that the functional term was not enriched in the indicated group. Other colors represent *P* values (Benjamini-Hochberg false discovery rate corrections). (**F**) Network of identified genes in the top enriched functional terms in each group. Nodes were marked out and labeled according to their functions in different biological processes. (**G**) Fingerprint plot of highlighted genes across the DDR-inhibitor screens. The genes shared by different groups are marked.

To determine the genetic vulnerabilities to DDR inhibition, we performed CRISPR screens in HEK293A cells with specific clinical/preclinical inhibitors (Figure 1A), including ATRi (AZD6738), ATMi (AZD0156), DNAPKi (NU7441) and CHK1i (LY2603618) (Figure 1B). These inhibitors targeted the PI3KK family kinases and a downstream CHK1 kinase, which are essential in DDR pathways. In this experimental setting, the mTOR inhibitor (rapamycin) was included as a control for the PI3KK family kinase inhibitors (Figure 1B) because mTOR is not directly involved in DDR. The CRISPR screens were carried out with the Toronto KnockOut single guide RNA (sgRNA) library (TKOv3) as previously described (43). The 20% inhibitory concentration (IC_20_) of each inhibitor was identified and shown to be effective in inhibiting the targeted kinase activity (Supplementary Figure S1). The CRISPR screen workflow is shown in Figure 1A. Briefly, cells were infected with the lentiviral sgRNA library and then were divided into control and treated groups. The treated groups were incubated with the indicated inhibitor at IC_20_ while the control groups were left untreated. After about 14 population doublings (∼21 days), genomic DNA extracted from the different groups were sequenced and analyzed. The gene-level depletion scores were analyzed with DrugZ, an optimized software application for drug-genomic CRISPR screens. The normalized *Z* scores (NormZ) are presented in Supplementary Table S1. Negative NormZ values suggest that depletion of the genes in question leads to synthetic weakness/lethality in the cell; positive NormZ values indicate genes whose loss confers a growth advantage in cells treated with the indicated drug.

We used the following criteria to obtain an overview of the screening results. For genes whose depletion induced sensitization to the DDR inhibitors, we used NormZ values less than –3 and synthetic lethal *P* values lower than 0.015 as the criteria for inclusion in a list of candidate genes whose loss could sensitize cells to each inhibitor. Our list of candidate genes included 468 genes for ATRi, 367 genes for CHK1i, 167 genes for ATMi, 135 genes for DNAPKi, and 89 genes for mTORi. This list was then processed for functional enrichment analysis for a global view of genetic vulnerabilities in response to individual inhibitors (Figure 1C, D).

A functional term enrichment analysis with STRING showed that, overall, these candidate genes are highly enriched in the functional categories associated with DNA replication, DNA repair, and cell cycle regulation. The top functional terms for each inhibitor are listed in Figure 1C. Specifically, we noticed great similarity between the top terms for ATRi and CHK1i (Figure 1C, D), which is as expected because they function in the same DDR pathway. Briefly our results showed that: (i) ATR activity is important for DNA replication and diverse DDR pathways, (ii) CHK1 activity is important for replication checkpoint control and cell cycle regulation, (iii) the loss of Fanconi anemia (FA) pathway genes increases sensitivity to ATMi, ATRi and CHK1i, and 4) defects in apurinic/apyrimidinic site (AP)-site resolution or the single-strand break repair (SSBR) pathway sensitize cells to DNAPKi. It is also worth noting that the functional terms enriched in the mTORi group are distinct, among which mTOR pathway genes were highly enriched, whereas the DDR-related terms rarely appeared. These functional enrichment terms were further analyzed by clustering, as shown in Figure 1E, which again shows the similarity between the pathways enriched by ATRi and CHK1i.

A relationship map of the individual genes in these diverse functional groups is presented in Figure 1F, which illustrates several important findings. First, ATRi and CHK1i shared the most common synthetic lethal/sick genes, most of which were cell cycle regulation-related genes. This finding was consistent with the findings from our functional enrichment analysis. Second, FA genes were highly enriched in both the ATMi and ATRi groups. Additionally, loss of genes involved in the base excision repair (BER) pathway sensitized cells to ATMi, ATRi and DNAPKi. This map also suggests preferences among these inhibitors. For example, many DNA replication-related genes were identified in the ATRi group, and some of these genes were shared with the ATMi or DNAPKi groups (Figure 1G), although the overlap was not extensive. The mTOR pathway genes in the mTORi group showed distinct signatures, as anticipated. It is also worth mentioning that we identified several genes with unclear functions that need further exploration.

We also compared our screen results with several recently reported screen datasets including ATMi based screen, ATRi based screen and CHK1i based screen (Supplementary Figure S2A and Table S2) (43–46). As shown in Supplementary Figure S2B-D, several robust interactions were recovered from different datasets although these screens were performed in different cell lines, with different inhibitors or with different sgRNA libraries. Several gene-drug interactions, e.g. the FA pathway with ATMi/ATRi, POLE3/4 with ATRi/CHK1i, USP37 with ATRi/CHK1i, BRCA1-A complex with ATMi, were recovered in all these screens (Supplementary Figure S2E), suggesting the reliability of CRIPSR screens in identifying synthetic interactions between genes and drugs. It is also of note that FA complex loss sensitized cells to ATMi/ATRi but not to CHK1i, while POLE3/4 loss sensitized cells to ATRi/CHK1i but not to ATMi. Also ATM loss sensitized cells to ATRi but protected cells from ATMi (Supplementary Figure S2E). These results suggest the similarities and differences among different DDR kinase inhibitors.

All these results suggest the reliability of these screens, the specificity of these DDR inhibitors, and the potential of this method for identifying novel gene-drug relationships that may improve the clinical application of these DDR agents.

### Loss of *YWHAE* Sensitizes Cells to CHK1i

**Figure 2.**
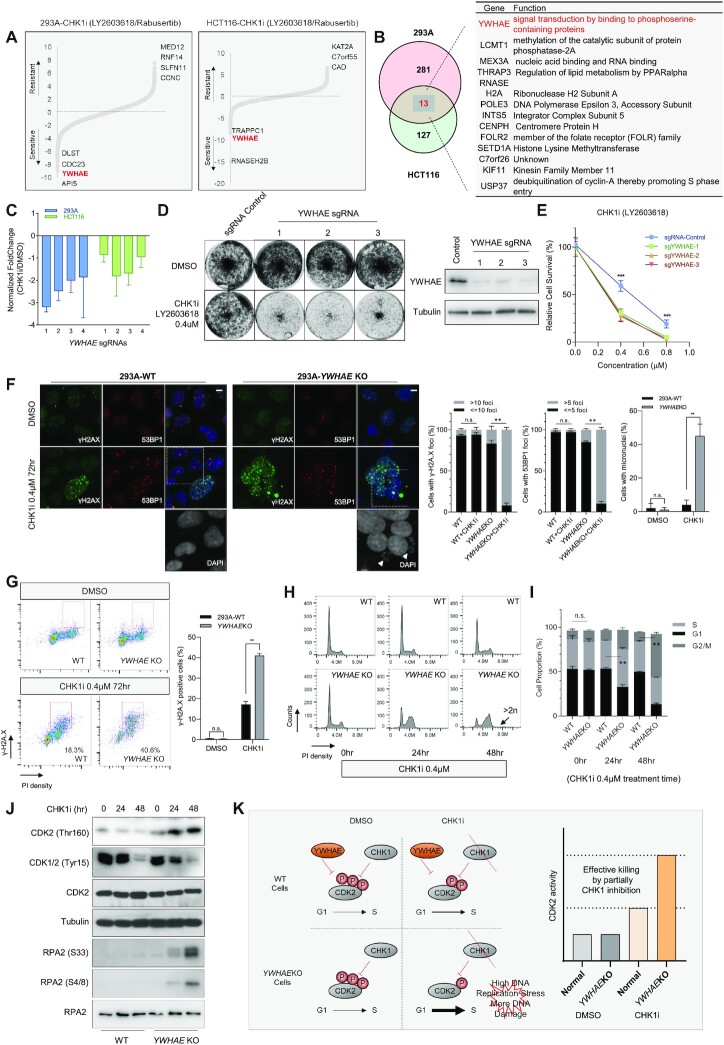
Loss of *YWHAE* Leads to Sensitivity to CHK1i. (**A**) Ranking of co-essential genes with CHK1i based on DrugZ analysis of CRISPR/Cas9-based screening results conducted in 293A and HCT116 cells. The NormZ score was used to define a possible synthetic lethal interaction with CHK1i. All genes targeted by the Toronto Knock Out Library (version 3) were scored according to the fold change of levels of their sgRNAs presented in these samples (CHK1i treatment vs. DMSO treatment). Genes whose loss of function led to sensitivity to CHK1i appear on the left side, and genes whose loss of function led to resistance to CHK1i appear on the right side. Some high-confidence genes are marked. (**B**) Venn diagram and list showing the overlapping CHK1i co-essential genes identified in these two cell lines. (**C**) Normalized fold changes of sgRNAs targeting YWHAE in the CHK1i group versus DMSO control group from the screens conducted in HEK293A and HCT116 cells. The fold change comes from the sgRNA counts in the CHK1i-treated group divided by the sgRNA counts in the DMSO group in the indicated cell lines. (**D**) Representative crystal violet cell viability assays with HEK293A cells under CHK1i treatment. HEK293A cells transfected with control sgRNA or sgRNAs targeting *YWHAE* were exposed to the indicated concentration of CHK1i inhibitors and grew for 7 days (1500 cells per well in 12-well plates). The efficacy of each sgRNA was validated by Western blotting. (**E**) Clonogenic survival assays at different CHK1i concentrations. The mean and s.d. of *n* = 3 independent experiments are shown, ***P < 0.001; Student's *t*-test. (**F**) Wild type (WT) and *YWHAE*-knockout (KO) HEK293A cells were treated with DMSO or CHK1i (0.4 μM, 72 h). Cells were fixed and processed for γ-H2A.X and 53BP1 immunofluorescence staining. The scale bar represents 1 μm. The nuclei were enlarged to highlight the accumulated micronuclei in CHK1i-treated *YWHAE*-KO cells. 100 cells/group were counted. The quantification of γ-H2A.X foci, 53BP1 foci, and micronuclei in different groups are graphed on the right. The mean and s.d. of *n* = 3 independent experiments are shown; n.s., not significant; **P < 0.01; Student's *t*-test. (**G**) WT and *YWHAE*-KO HEK293A cells were treated with DMSO or CHK1i (0.4 μM, 72 h). Cells were fixed and processed for γ-H2A.X and propidium iodide staining. Fluorescence-activated cell sorting analyses were then conducted. The red rectangle indicates the γ-H2A.X-positive cells. For γ-H2A.X staining, 30 cells/group were counted. Quantification of γ-H2A.X-positive cells in each group is shown on the right. The mean and s.d. of *n* = 3 independent experiments are shown; n.s., not significant; **P < 0.01; Student *t*-test. (**H**) WT and *YWHAE*-KO HEK293A cells were treated with DMSO or CHK1i. The cells were then collected at different time points, fixed with ethanol, and stained with propidium iodide. FACS analyses were then conducted. (**I**) Quantification of cell populations in different cell cycle phases in WT and *YWHAE*-KO cells. The indicated cell populations were compared between the WT group and the *YWHAE*-KO group under CHK1i treatment. The mean and s.d. of *n* = 3 independent experiments are shown, **P < 0.01; Student's *t*-test. (**J**) The same cells in (**I**) were collected. Total cell lysates were blotted with the indicated antibodies. (**K**) A proposed model of hypersensitivity to CHK1i in *YWHAE*-KO cells.

As mentioned above, we found that CHK1i and ATRi share a catalog of synthetic lethal/sick genes that are known to be involved in cell cycle regulation. CHK1i has been less thoroughly studied than ATRi; therefore, there is a pressing need to identify the determinants of sensitivity to CHK1i. To achieve a comprehensive view of CHK1i's synthetic lethality in cells with diverse genetic backgrounds, we performed an additional CRISPR screen with the same CHK1i in HCT116 cells (Figure 2A). We identified 13 common genes whose loss caused sensitivity to CHK1i in both cell lines. Among the genes showing synthetic lethality with CHK1i, *YWHAE* scored as one of the top candidate genes (Figure 2B).

*YWHAE* belongs to the 14–3–3 family of genes. The 14–3–3 family proteins are highly conserved in species from yeast to humans and in mammals consist of seven mammalian genes/isoforms (β, γ, ζ, η, θ, σ and ϵ) (47). The 14–3–3 family proteins have different expression patterns in different cell types and tissues (48,49). Through binding to diverse target proteins, the 14–3–3 family proteins regulate multiple biological processes such as cell proliferation, cell survival, apoptosis, and stress signaling (49,50). Given the structural similarity between the isoforms, 14–3–3 family proteins have overlapping yet distinct functions (48). To evaluate the specificity of *YWHAE* loss in inducing sensitization to CHK1 inhibition, we compared the sgRNA dropout data of these 14–3–3 family genes. As shown in Figure 2C and Supplementary Figure S3A, the counts of the sgRNAs targeting YWHAE, but not those targeting other 14–3–3 isoforms, decreased significantly under treatment with CHK1i, which suggests that YWHAE depletion specifically leads to hypersensitivity to CHK1i. We further validated the synthetic lethal interaction between YWHAE and CHK1i using clonogenic experiments. As shown in Figure 2D and E and Supplementary Figure S3B, transfection with different sgRNAs reduced YWHAE protein levels and significantly enhanced sensitivity to CHK1i in both HEK293A and HCT116 cells, which confirmed our CRISPR screen results.

To search for the mechanisms underlying the synthetic lethality between *YWHAE* and CHK1, we compared the responses of HEK293A-wild type (WT) and HEK293A-*YWHAE*-knockout (KO) cells to CHK1 inhibition. As shown in Figure 2F, both γ-H2A.X signals and 53BP1 foci increased dramatically in *YWHAE*-KO cells, suggesting that more DNA damage accumulated in these KO cells under CHK1i treatment. Moreover, the number of cells with micronuclei also increased significantly in CHK1i-treated *YWHAE*-KO cells compared to control WT cells (Figure 2F), which again may be the consequence of DNA damage accumulated in those cells. Fluorescence-activated cell sorting (FACS) analysis further confirmed the increased γ-H2A.X signals and the higher G2/M cell population in CHK1i-treated *YWHAE*-KO cells (Figure 2G). Although we noticed augmented dead cell population in CHK1i treated *YWHAE*-KO cells (Supplementary Figure S3D), we did not observe any significant increase in apoptotic cells, i.e. the annexin V positive cell population, suggesting that the upregulated γ-H2A.X signals were unlikely due to any increase in apoptosis. All these results indicate that accumulation of DNA damage is one reason that *YWHAE* loss induces hypersensitivity to CHK1i.

Because YWHAE has previously been associated with cell cycle regulation through its regulation of CDC25A/B/C activities (51), we hypothesized that the accumulation of DNA damage may be caused by dysfunctional cell cycle regulation in *YWHAE*-KO cells. Indeed, we noticed that under CHK1i treatment, the number of S-phase cells was higher in the YWHAE-KO population than in the WT population. As shown in Figure 2H-I and Supplementary 3E, after 24 h of CHK1i treatment, we observed a significantly increased S-phase cell population in *YWHAE-*KO cells and an increased G2/M fraction. These changes became more dramatic after 48 h of CHK1i treatment. These results indicate that *YWHAE*-KO cells suffer prolonged S/G2 progression in the presence of CHK1i. We also observed a lower pCDK1/2-Tyr15 level, a higher pCDK2-Thr160 level, and a higher pRPA2-S33/pRPA2-S4/8 level in CHK1i-treated *YWHAE*-KO cells than those in WT cells (Figure 2J). We further examined the replication fork dynamics in WT and *YWHAE*-KO mutant cells under CHK1 inhibition using DNA fiber assay. CHK1i treatment had no detectable impact on replication speed in WT cells but reduced replication speed in *YWHAE*-KO cells (Supplementary Figure S3F and G), which suggests more replication stress in CHK1i-treated YWHAE-KO cells. All the results above suggest that hyper activation of CDK1/2 in *YWHAE*-KO cells under CHK1i may enhance replication stress and eventually lead to an accumulation of DNA damage and genomic instability under CHK1i treatment (Figure 2K).

Furthermore, as shown in Supplementary Figure S3H, a CDK2 inhibitor CVT313 could partially rescue the cytotoxicity of CHK1i in both WT and *YWHAE* KO cells, which is consistent with our working hypothesis (Figure 2K). Moreover, the CRISPR screen results suggest that YWHAE loss could also sensitize cells to ATRi (Supplementary Figure S2E), which was further confirmed by clonogenic assay (Supplementary Figure S3I). As ATR also participate in cell cycle checkpoint regulation, these results further support our hypothesis presented in Figure 2K.

### *KLHL15* loss protects cells from ATMi-induced DNA damage

**Figure 3.**
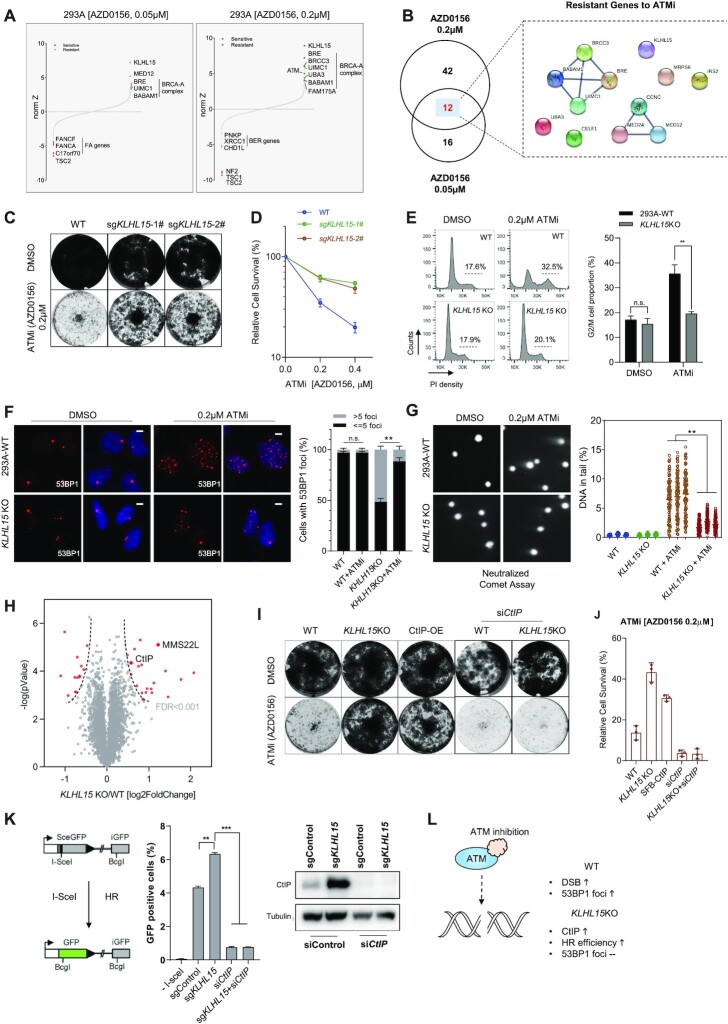
*KLHL15* loss protects cells from ATMi-induced DNA damage. (**A**) Ranking of ATMi co-essential genes after DrugZ analysis based on the results of CRISPR/Cas9-based screening using two different concentrations of ATMi. The NormZ score was used to define a possible synthetic lethal interaction with ATM inhibition. Genes whose loss of function led to ATMi sensitivity appear on the left side, and genes whose loss of function led to ATMi resistance appear on the right side. Some high-confidence genes are marked (red for synthetic lethal genes, green for resistant genes). (**B**) Venn diagram showing the overlapping of identified genes responsible for resistance to ATMi. The genes that overlapped in two different ATMi concentrations are listed and the gene–gene network was drawn according to STRING. (**C**) Loss of *KLHL15* made cells resistant to ATMi treatment based on crystal violet cell viability assays. HEK293A cells transfected with control sgRNA or sgRNAs targeting *KLHL15* were exposed to the indicated concentrations of ATMi and grew for 7 days (1500 cells per well in 12-well plates). (**D**) Clonogenic survival assays at the indicated concentrations of ATMi. The mean and s.d. of *n* = 3 technical replicates are shown. (**E**) Wild type (WT) and *KLHL15*-knockout (KO) HEK293A cells were treated with DMSO or ATMi. Cells were then collected after 3 days, fixed with ethanol, and stained with propidium iodide. Fluorescence-activated cell sorting analyses were then conducted. The indicated G2/M cell populations in each group were compared. The mean and s.d. of *n* = 3 independent experiments are shown; n.s. = not significant; **P < 0.01; Student's *t*-test. (**F**) WT and *KLHL15*-KO HEK293A cells were treated with DMSO or ATMi (AZD0156, 0.2 μM, 72 h). Cells were fixed and processed for 53BP1 immunofluorescence staining. The scale bar represents 1 μm. 100 cells/group were counted. Quantification of 53BP1 foci in the indicated groups is shown on the right. The mean and s.d. of *n* = 3 independent experiments are shown; **P *<* 0.01; n.s. = not significant; Student's *t*-test. (**G**) Representative images from a neutralized comet assay. WT and *KLHL15*-KO HEK293A cells were exposed to ATMi (AZD0156, 0.2 μM) for 24 h. The comet-tail moments from 100 cells in each condition were measured and are shown in the scatter plot. The mean and s.d. of *n* = 3 independent experiments are shown; **P < 0.01; Student's *t*-test. (**H**) Volcano plot shows the significant change in differentially expressed proteins in *KLHL15* KO versus WT cells. Each dot represents a protein. The red dots indicate significantly upregulated and downregulated proteins, respectively (FDR < 0.001). y axis is the –log *P*-value, and x axis is the log_2_ fold change. Two known DDR related proteins are labeled with gene names. (**I**) Crystal violet viability assays of the indicated cells. HEK293A cells were transfected with the indicated constructs or siRNA and then exposed to the indicated ATMi (AZD0156) treatment for 7 days (1500 cells per well in 12-well plates). Abbreviations: OE, overexpressing. (**J**) Results of clonogenic survival assays at the indicated concentrations of ATMi (AZD0156). The mean and s.d. of *n* = 3 technical replicates are shown. (**K**) Loss of *KLHL15* resulted in increased HR-directed DNA repair. U2OS-DR-GFP cells were infected with lentivirus-encoding control sgRNA or sgRNA targeting *KLHL15* together with/without *CtIP* siRNA. The cells were then transfected with I-SceI; 48 h after transfection, cells were harvested and assayed for GFP expression using FACS. Representative data from one experiment are shown. The GFP-positive cells were gated. Quantification of these experiments is shown on the right side of the panel. The mean and s.d. of *n* = 3 independent experiments are shown; **P < 0.01; Student's *t*-test. The expression levels of CtIP were detected by Western blotting. (**L**) Proposed model of KLHL15 depletion leading to CtIP upregulation and resistance to ATMi.

While mining the ATMi screen results (Figure 3A), we found that loss of FA pathway components sensitized cells to ATMi, as previously reported (44). Additionally, we found that loss of BRCA-A complex genes may cause resistance to ATMi. More excitingly, *KLHL15* was identified as the top gene whose loss induced dramatic resistance to ATMi (Figure 3A). To further validate these findings, we repeated the CRISPR screen using a higher concentration of ATMi (Figure 3A), which again showed significant enrichment of BRCA-A complex genes and *KLHL15* as the top ATMi resistance-associated genes. We then validated these results and showed that, compared to WT cells, cells transfected with different sgRNAs targeting *KLHL15* displayed significant resistance to ATMi (Figure 3C and D). We further created and validated *KLHL15*-KO cells by genomic sequencing (Supplementary Figure S4A) for the following experiments.

Cells treated with ATMi alone displayed an increased G2/M population (Figure 3E), indicating that ATMi treatment may cause accumulation of DNA damage. Interestingly, in *KLHL15-*KO cells, the G2/M arrest induced by ATMi was significantly reduced (Figure 3E), indicating reduced DNA damage in these cells. These results were further confirmed by analysis of 53BP1 foci, which were induced by ATMi in WT cells but dramatically suppressed in *KLHL15-*KO cells (Figure 3F). Moreover, a neutral comet assay revealed that ATMi treatment induced DNA double-strand breaks in control WT cells, but these breaks were significantly reduced in *KLHL15-*KO cells (Figure 3G). Collectively, these data validated the CRISPR screen results and suggested that ATMi-induced DNA damage may be efficiently repaired in *KLHL15-* KO cells and therefore result in ATMi resistance. As KLHL15 is a component of the E3 ligase complex, we profiled the KLHL15KO cells using TMT-proteomics. As shown in Figure 3H and Supplementary Table S3, the levels of several proteins were significantly increased (FDR < 0.001), including two DNA repair related proteins (C-terminal interacting protein [CtIP] and MMS22L). KLHL15 has been reported as a component of the E3 ligase complex that targets C-terminal interacting protein (CtIP) for degradation (52). Since we observed dramatic CtIP protein level increases in KLHL15-KO cells (Supplementary Figure S4B), we reasoned that CtIP upregulation may account for ATMi resistance in these cells. As shown in Figure 3I, J and Supplementary Figure S4B, similar to *KLHL15-*KO, overexpression of CtIP also resulted in resistance to ATMi in 293A cells. Moreover, when we knocked down CtIP expression in *KLHL15*-KO cells, cells regained their sensitivity to ATMi treatment (Figure 3I, J). As CtIP plays an important role in HR-mediated double-strand break repair (53), we also checked the HR efficiency in *KLHL15*-depleted cells and confirmed that *KLHL15* loss increased HR capability (Figure 3K). All these results suggest that *KLHL15*-KO-induced ATMi resistance may come from the elevated HR efficiency associated with increased CtIP expression and function (Figure 3L).

It is also noteworthy that our findings showed that the loss of the BRCA1-A complex (except BRCA1) also led to ATMi resistance (Figure 3A, B and Supplementary Figure S2E), although the loss of the BRCA1-A complex had a slightly weaker effect than the loss of *KLHL15*. This finding is consistent with that of a previous report (44). On the other hand, we found that loss of BRCA1 or CtIP led to increased sensitivity to ATMi (Supplementary Table S1). As loss of components in BRCA1-A complex has previously been reported to increase HR efficiency (54,55), we speculate that HR capability is a critical determinant of cellular response to ATMi. Indeed, we showed that, similar to CtIP, reducing BRCA1 expression also resensitized *KLHL15*-KO cells to ATMi (Supplementary Figure S4B, C, D). Moreover, similar to *CtIP* knockdown, *BRCA1* knockdown reduced the HR efficiency in *KLHL15*-KO cells (Supplementary Figure S3E).

Additionally, we validated that loss of BRCA1-A complex led to ATMi resistance. As shown in Supplementary Figure S3F, *RAP80*-KO cells were more resistant to ATMi which could be further rescued by *BRCA1* knock-down. Our results suggest that competition between the BRCA1-A complex and the BRCA1-CtIP complex may regulate the HR process, as previously reported (54,55). The detailed relationship between these BRCA1-containing protein complexes warrants further investigation.

### ATM is necessary for the cell proliferation inhibition induced by ATMi

**Figure 4.**
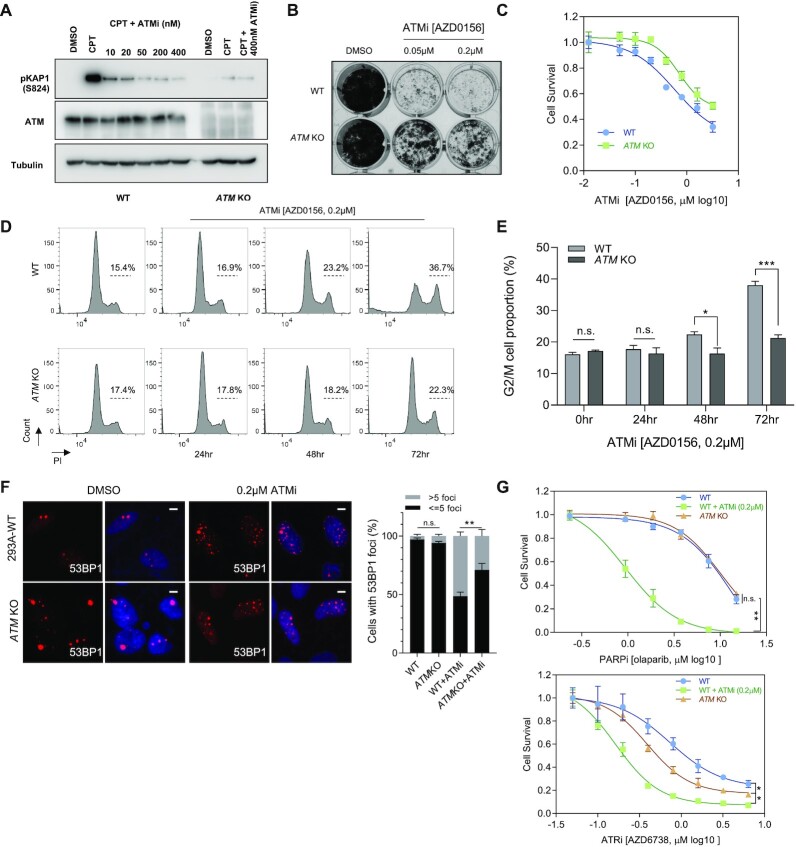
ATM is required for the Cytotoxicity of ATM inhibition. (**A**) Validation of the *ATM*KO cells. The HEK293A WT cells or *ATM*KO cells were treated with CPT (100 nM, 3 h) together with/without ATMi-AZD0156 (0.01–0.4 μM). Cell lysates were collected. Western blotting was conducted with the indicated antibodies. (**B**) Loss of *ATM* made cells resistant to ATMi treatment in crystal violet cell viability assays. The HEK293A WT cells or *ATM*KO cells were exposed to the indicated concentrations of ATMi (AZD0156) and grew for 7 days (1500 cells per well in 12-well plates). The results are representative of three independent experiments. (**C**) Dose–response survival curves of HEK293A-WT, HEK293A-*ATM* KO exposed to increasing concentration of ATMi. Cells were treated with ATMi under different concentrations for 3 days and the cell survival was measured with cell-titer glo assay. The mean and s.d. of *n* = 3 technical replicates are shown. (**D**) WT and *ATM*-KO HEK293A cells were treated with DMSO or ATMi (AZD0156). The cells were then collected after 3 days, fixed with ethanol, and stained with propidium iodide. Fluorescence-activated cell sorting analyses were then conducted. (**E**) Quantification of the differences between the indicated G2/M cell populations in each group are presented. The mean and s.d. of *n* = 3 independent experiments are shown; n.s. = not significant; ***P < 0.001, *P< 0.05; Student t-test. (**F**) WT and *ATM*-KO HEK293A cells were treated with DMSO or ATMi (AZD0156, 0.2 μM, 72 h). Cells were fixed and processed for 53BP1 immunofluorescence staining. These experiments were performed in parallel with those shown in Figure 3F. The scale bar represents 1 μm. 30 cells/group were counted. Quantification of 53BP1 foci in the indicated groups is shown on the right. The mean and s.d. of *n* = 3 independent experiments are shown; **P < 0.01; n.s. = not significant; Student's t-test. (**G**) WT and *ATM*-KO HEK293A cells were treated with DMSO or ATMi (AZD0156, 0.2 μM). The cells were then exposed to increasing concentration of PARPi (upper) or ATRi (lower) for 3 days. The cell survival was measured with cell-titer glo assay. The mean and s.d. of *n* = 3 independent experiments are shown, *P value < 0.05; **P value < 0.01; n.s. = not significant; Student t-test.

The CRISPR screen results also suggest ATM loss could protect cells from ATMi (Supplementary Figure S2E and Figure 3A), which was consistent with a previous report (44). However, the underlying mechanism is not well studied. We firstly validated this result using *ATM*KO in HEK293A cells. Cells were treated with CPT (100 nM) without or with ATMi-AZD0156 (0.01–0.4 μM) for 3 h. In WT cells, ATMi inhibited CPT-induced ATM signaling in a dose-dependent manner as measured by phosphorylation of an ATM substrate KAP1 (Ser-824). In *ATM*KO cells generated with CRISPR, the phosphorylation of KAP1 (Ser-824) was almost depleted (Figure 4A). These results indicated that inhibition by ATMi phenocopies the ATM knockout model.

In clonogenic assay, *ATM*KO cells were significant resistant to ATMi treatment when compared to WT cells (Figure 4B). The cell-titer glo assay further demonstrated that *ATM*KO cells were more resistant to ATMi (Figure 4C). Moreover, as shown in Supplementary Figure S5A, *ATM*KO cells were more resistant to a different ATMi AZD1390. These results, together with the CRISPR screen results based on another ATMi M3541 (44), suggest the synthetic interaction between *ATM*KO and ATMi was not limited to one ATMi. As shown in Figure 4D and E, extended treatment with ATMi induced increased G2/M population in control cells, but this G2/M accumulation induced by ATMi was significantly reduced in *ATM*KO cells, indicating that ATMi may induce less DNA damage in *ATM*KO cells. These results were further confirmed by analysis of 53BP1 foci, which were induced by ATMi in WT cells but suppressed in *ATM*KO cells (Figure 4F). All these observations suggest that ATM protein is necessary for G2 accumulation and cell proliferation inhibition induced by ATMi. Thus, we conclude that the ATMi induced cell proliferation inhibition is due to the generation of inactive ATM, which may show dominant negative effect in the cell.

We then checked the differences between ATM inhibition and *ATM*KO under the treatment of other DDR inhibitors. As shown in Figure 4G, ATMi (AZD0156) treatment dramatically sensitized cells to PARPi treatment while *ATM*KO did not. Moreover, a different ATMi (AZD1390) also significantly sensitized cells to PARPi treatment (Supplementary Figure S5C), which further confirmed the synergistic effect of ATMi and PARPi. Furthermore, ATM KO could partially rescue the synergistic effect induced by ATMi + PARPi (Supplementary Figure S5C). Additionally, *ATM* knock-down did not sensitize cells to PARPi treatment in HEK293A cells (Supplementary Figure S5D), which is consistent with the observation of PARPi treatment in *ATM*KO cells. It is also of note that, although both ATMi (0.05 μM) treatment and *ATM*KO sensitized cells to ATRi treatment, ATMi treatment led to a higher ATRi sensitivity than that of *ATM*KO (Figure 4G). All these results suggest inactive ATM induced by ATM inhibition abrogates DNA repair more than ATM loss.

### *APEX1* loss sensitizes cells to DNAPK inhibition

**Figure 5.**
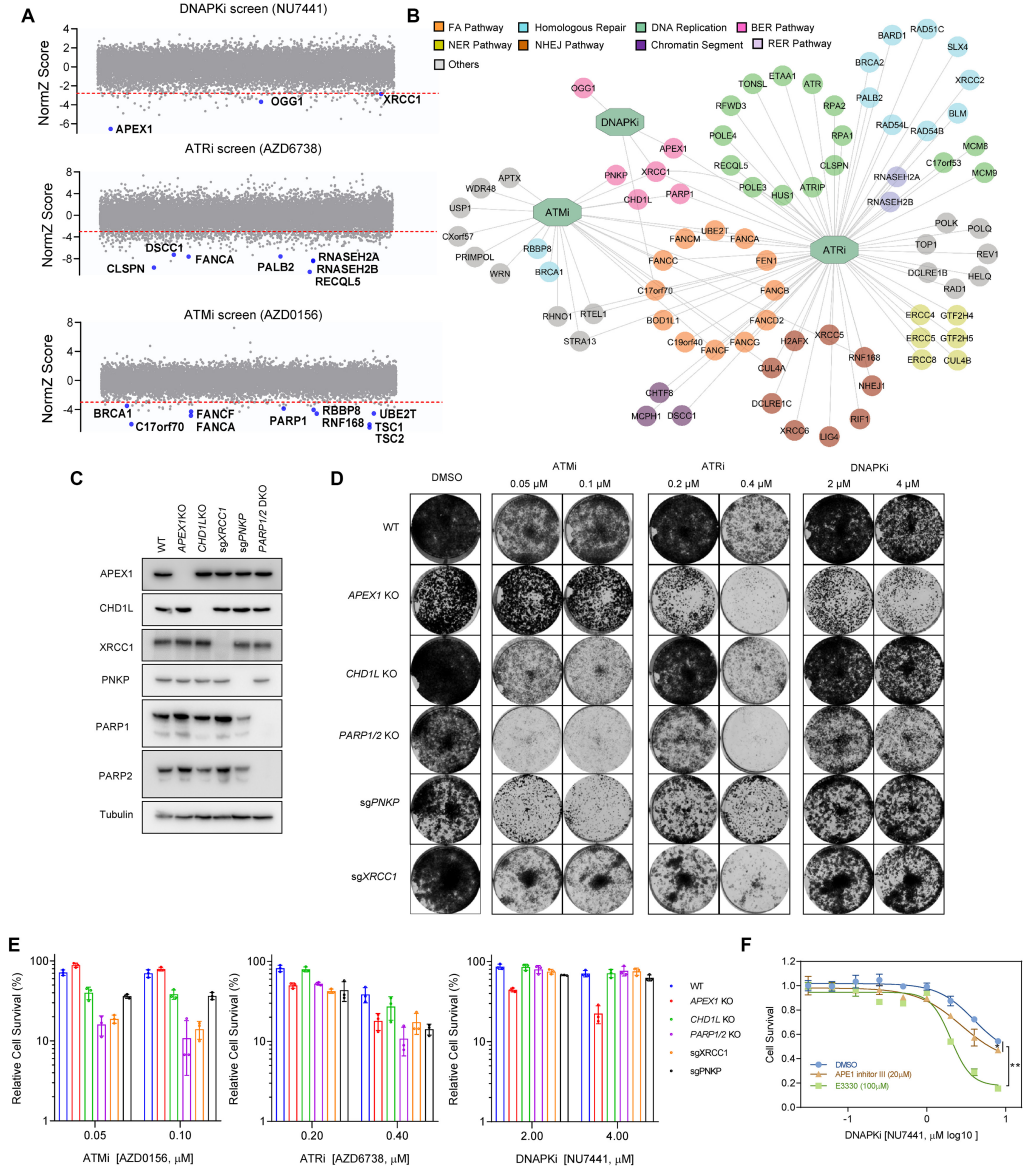
*APEX1* Loss Sensitizes Cells to DNAPK Inhibition. (**A**) CRISPR dropout screen results for HEK293A cells exposed to DNAPKi, ATMi and ATRi. Selected DDR-related genes showing synthetic lethality with each of these inhibitors are marked. (**B**) Network of identified genes in the curated DDR gene list from each group. Nodes were marked and labeled according to their functions in different DDR processes. (**C**) Validation of depletion of BER proteins in HEK293A cells. The indicated knockout (KO) cells or cells infected with the indicated sgRNAs were collected. Cell lysates were prepared and analyzed by Western blotting with the indicated antibodies. (**D**) Crystal violet viability assays of the indicated cells. The indicated cells were exposed to the indicated inhibitors at the indicated concentrations for 7 days (1500 cells per well in 12-well plates). (**E**) Results of clonogenic survival assays at the indicated concentrations of the inhibitors. The mean and s.d. of *n* = 3 technical replicates are shown. (**F**) WT HEK293A cells exposed to increasing concentration of E3330 or APE1 inhibitor III for 3 days. Cell survival was measured with cell-titer glo assay. The mean and s.d. of *n* = 3 independent experiments are shown, *P value < 0.05; **P value < 0.01; ANOVA test.

To get insights into how the loss of distinct DNA repair genes affects cellular sensitivities to inhibitors targeting DDR-related PI3KK family members, we compared the dropout numbers of a curated set of DDR genes in response to each inhibitor. As shown in Figure 5A and B, our screen results demonstrate that ATR activity is critically important for the survival of cells with defects in a variety of DNA replication and DDR pathways, such as HR, NHEJ, nucleotide excision repair (NER), and BER pathways. These results further confirmed the versatile roles of ATR in DDR. Additionally, ATM activity is important to the survival of cells with defective FA, HR, and other DDR pathways. As a matter of fact, the FA pathway was enriched in both the ATRi and ATMi groups, suggesting that both ATR and ATM are needed for the survival of FA cells. Compared to the CRISPR screens with ATRi and ATMi, the screen with DNAPKi identified fewer DDR-related genes, implying that DNAPK may have limited functions in DDR or that it shares redundant functions with ATM and/or ATR. These genetic interactions in response to DDR inhibitors revealed the diverse and overlapping functions of these PI3KK kinases in DDR.

We noticed that the genes whose loss led to cellular sensitivity to all three PI3KK kinase inhibitors belong to BER/SSBR pathway (Figure 5B). We then explored the synthetic lethal relationships between BER genes and DDR inhibitors. Specifically, we depleted different BER genes (e.g. *XRCC1, CHD1L, PNKP, PARP1/2, APEX1*) in cells (Figure 5C) and tested their sensitivities to ATRi, ATMi, or DNAPKi. As shown in Figure 5D, E, the loss of several components involved in the BER/SSBR pathway, e.g. *XRCC1*, *PNKP*, *CHD1L* and *PARP1/2*, led to hypersensitivity to ATRi and ATMi but not to DNAPKi. *APEX1* loss led to hypersensitivity to DNAPKi and ATRi but not to ATMi. These data further confirmed our screening results.

Only few DDR genes when depleted showed hypersensitivity to DNAPKi. Among them, *APEX1* scored as the top synthetic lethal gene (Figure 5A). APEX1 functions in both redox signaling and DNA glycosylation (56). To explore whether both redox activity and AP endonuclease activity of APEX1 are important for DNAPKi sensitivity, we checked the synergistic effects between DNAPKi and the inhibitors separately targeting APEX1 redox activity (E3330) or AP endonuclease activity (APE1 inhibitor III). As shown in Figure 5F, both E3330 treatment and APE1 inhibitor III treatment could sensitize cells to DNAPKi. Notably, the inhibition of APEX1 redox activity showed stronger synergistic effect with DNAPKi. These results suggest that both the redox activity and AP endonuclease activity of APEX1 are necessary for the survival of DNAPKi treated cells.

### The combination of ATMi and PARPi induces apoptosis

**Figure 6.**
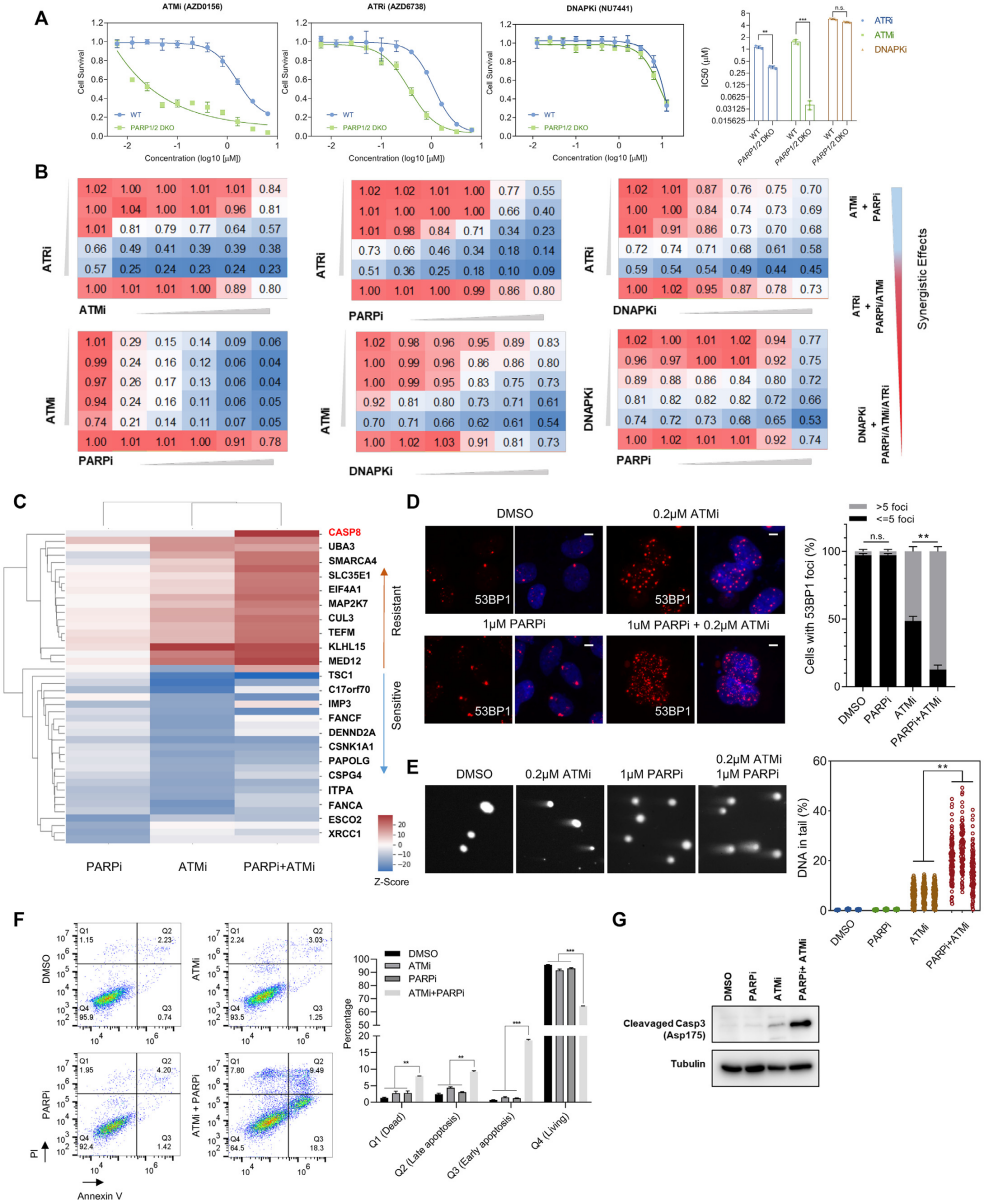
Comparison of synergistic effects of different DDR inhibitor combinations. (**A**) Representative dose-response survival curves of HEK293A wild type (WT) or PARP1/2-knockout (KO) cells exposed to increasing concentrations of ATMi, ATRi or DNAPKi. The mean and s.d. of *n* = 3 technical replicates are shown. The IC_50_ value of each inhibitor in different cells is shown on the right side of the panel (The mean and s.d. of *n* = 3 independent experiments are shown). ***P < 0.001; **P < 0.01; n.s. = not significant; Student's *t*-test. (**B**) Results of synergistic assays of different drug combinations. Cells were treated with increasing doses of ATRi (AZD6738; 0–1.0 μM), ATMi (AZD0156; 0–0.8 μM), DNA-PKi (NU7441; 0–4 μM), or PARPi (olaparib; 0–4 μM). The numbers denote cell viability relative to cells given control DMSO treatment. Color ranges: red (100% viability) to blue (0% cell viability). (**C**) Comparisons for the determinant sensitivity and/or resistance genes from the CRISPR screens (ATMi, PARPi, ATMi plus PARPi) are shown. Color ranges: red (resistant) to blue (sensitive). (**D**) HEK293A cells were treated with DMSO, ATMi, PARPi or ATMi plus PARPi for 24 h. Cells were fixed and processed for 53BP1 immunofluorescence staining. These experiments were performed in parallel with those shown in Figures 3F and 4F. The scale bar represents 1 μm. 30 cells/group were counted. Quantification of 53BP1 foci in different groups is shown on the right. The mean and s.d. of *n* = 3 independent experiments are shown; n.s. = not significant; **P < 0.01; Student's *t*-test. (**E**) Representative images from neutral comet assay. HEK293A cells were treated with DMSO, ATMi 0.2μM, PARPi 1 μM or ATMi 0.2 μM plus PARPi 1 μM for 24 h and then processed for the comet assay. These experiments were performed in parallel with those shown in Figure 3G. The comet-tail moments from more than 100 cells in each group were measured and are shown in the scatterplot. The mean and s.d. of *n* = 3 independent experiments are shown; **P < 0.01; Student *t*-test. (**F**) Flow cytometry analysis of cell death of HEK293A WT cells under different treatments (ATMi 0.2μM, PARPi 1 μM, ATMi 0.2μM plus PARPi 1 μM). Cells were treated as indicated for 3 days. Cells were then stained with Annexin-V-FITC and PI, which were further analyzed by FACS. Cells were distinguished into different groups: Q1-Dead cells, Q2-Late Apoptosis cells, Q3-Early apoptosis cell, Q4-Living cells. Representative results were shown in the left panel. Quantification of the experiments were shown in the right panel. The mean and s.d. of *n* = 3 independent experiments are shown, *n* = 3. ***P < 0.001, **P < 0.01, n.s. = no significant change, Student's *t*-test. (**G**) The same cells in (F) were collected and whole cell lysates were blotted with the indicated antibodies.

The overlapping functions of these inhibitors and the data presented above suggest potential synthetic lethality between *PARP1/2* and these DDR inhibitors. We further determined these genetic interactions using *PARP1/2* double KO cells. As shown in Figure 6A, *PARP1/2* double KO cells were hypersensitive to ATMi and ATRi but not to DNAPKi. These data agree with the results shown above in Figure 5 and prompted us to compare the synergistic effects of combining different DDR inhibitors, including PARPi, ATMi, ATRi and DNAPKi. 293A cells in 96 well plates were treated with different drug combinations and cell variabilities were tested using cell-titer glo after 72 h treatments. As shown in Figure 6B, the ATMi plus PARPi combination showed the highest synergistic effect, followed by ATRi plus PARPi and ATMi plus ATRi. The combinations of DNAPKi and other inhibitors did not display any synergistic effects. All these data further validated our screen results and provided a quantitative view of the effects of combining these four different DDR-targeting agents.

To determine how the combination of ATMi plus PARPi caused dramatic cytotoxicity, we performed CRISPR screens with PARPi plus ATMi as well as with PARPi only and compared these results with those for ATMi (Figure 6C). Genes whose loss caused resistance to the ATMi plus PARPi combination included *KLHL15* and components of the BRCA1-A complex; this finding was consistent with our CRISPR screen results for ATMi only (Figure 3). Interestingly, we determined that *CASP8* was the top resistance-associated gene in the PARPi plus ATMi group but not in the PARPi or ATMi single-agent groups. We suspected that the synergistic effect of the ATMi plus PARPi combination may be due to apoptosis induced by excess DNA damage. Indeed, 53BP1 foci and double-strand breaks increased dramatically under treatment with ATMi plus PARPi (Figure 6D, E). Moreover, the annexin V positive cell population (Figure 6F) or the cleaved Caspase-3 level (Figure 6G) was significantly increased in ATMi plus PARPi treated group. Collectively, these results suggest that treatment with ATMi plus PARPi causes double-strand break accumulation, which may further induce cell death via apoptosis.

## DISCUSSION

In this study, we performed systematic screens to identify genetic vulnerabilities under treatment with several well-established DDR inhibitors. Our results not only revealed genetic vulnerabilities in response to these DDR inhibitors, but also permitted comparisons between these results, which further improve our understanding of the common and specific functions of these DDR inhibitors.

We also compared our screen results with other CRISPR screen datasets and recovered many robust synthetic interactions, such as FA complex with ATMi/ATRi, POLE3/4 with ATRi/CHK1i, USP37 with ATRi/CHK1i, BRCA1-A complex with ATMi, which further confirmed the reliability of CRISPR screens for the identification of gene–drug interactions. It is also of note that the small overlaps between the results derived from different cell lines/inhibitors/sgRNA libraries suggest that synthetic interactions may vary due to different genetic backgrounds/different characteristics of chemicals/sgRNA library applied. Additionally, we compared the results derived from cells treated with different dosages of ATMi and found that the screen with high dosage may perform better in identifying synthetic survival interactions while the screen with low dosage may keep more synthetic lethal interactions. It is also of note that the overlap of synthetic lethal genes between the screens with two different concentrations of ATMi is limited, although the top synthetic survival hits are largely the same. One possible reason is that treatment with higher dosage of ATMi may induce extra DNA damage since more dominant negative form of ATM were generated following ATMi treatment. Indeed, we showed that treatment with higher dosage of ATMi led to severe G2/M accumulation, which could be rescued by ATM KO. As a result, ATM was readily identified as a synthetic survival hit in the screen with higher dosage of ATMi. We reasoned that several synthetic lethal genes identified in the screen with higher dosage of ATMi may be indispensable to counteract extra DNA damage induced by dominant negative form of ATM, which needs further investigation. Of course, we cannot exclude other possibilities since high dosages of ATMi could inhibit cell proliferation even in ATM KO cells.

Our results showed that, as expected, CHK1i and ATRi share extensive similarities. Compared to the screens for other DDR inhibitors, we uncovered more genes involved in cell cycle regulation in the CHK1i/ATRi screens, which further confirmed the diverse functions of the ATR-CHK1 pathway in cell cycle regulation. Specifically, by comparing the CHK1i screen results in different cell lines, we identified *YWHAE* as a major determinant of sensitivity to CHK1 inhibition. Our follow-up experiments suggest that abnormal activation of cyclin-dependent kinase 2 (CDK2) activity caused by *YWHAE* loss contributes to sensitivity to CHK1i. This finding is consistent with that of previous reports suggesting that CDK2 activity determines CHK1i efficacy (57,58). Notably, several in-development CHK1 inhibitors also target CDKs, for instance MK-8776, which may reduce the efficacy of CHK1i by inhibiting CDKs (59). Thus, the combination of CDK and ATR/CHK inhibitors is unlikely to be beneficial in clinic. Moreover, as *YWHAE* loss has been reported in gastric cancers and is associated with proteasome-inhibitor resistance in multiple myeloma (60,61), CHK1i treatment may be a viable option for patients with these cancers. Future studies are needed to further substantiate this hypothesis, which may provide a key biomarker for CHK1i-based therapy.

Our screens with ATMi identified respectively the FA and BRCA1-A complexes as determinants of sensitivity and resistance to ATMi; this finding is consistent with that of previous reports (44). Moreover, we discovered that the loss of *KLHL15* leads to dramatic ATMi resistance. We further demonstrated that the loss of *KLHL15* induces CtIP upregulation and increases HR efficiency, which may at least in part accounts for ATMi resistance in this setting. Moreover, our results provide hints of competition between the BRCA1-A and BRCA1-CtIP complexes in HR regulation, which warrants further investigation.

We also found and validated ATM as a determinant of resistance to ATMi which is consistent with a previous report (44). The underlying mechanism was poorly studied although loss of a drug target is a common cause of acquired drug resistance (62). Here, we compared the responses of *ATM*KO and ATMi to different DDR inhibition and found ATMi abrogates DNA repair more than that of ATM loss. These findings are consistent with several recent reports (63,64). As it was reported previously that the kinase dead ATM protein caused embryonic lethality and more genomic instability than ATM loss (65,66), we propose that the underlying mechanism is that ATMi induces ATM into a dominant negative form, which is more cytotoxic than the loss of ATM protein. It is of note that *ATM* KO did not significantly sensitize cells to PARPi in HEK293A cells, which is consistent with a recent report showing that ATM KO did not enhance PARPi efficacy in 3 prostate cancer cell lines (63). However, ATM loss was reported to sensitize several other cancer cells to PARPi (67,68). Different genetic backgrounds may partially account for these differences, which need to be further investigated. Nevertheless, based on our observations and the findings by others (63), the current working hypothesis is that PARPi such as olapraib may not show significant clinical benefits for cancer patients with loss of ATM protein. Future clinical studies are needed to test this hypothesis directly.

Interestingly, the BER pathway turned out to be a critical pathway, the loss of which was associated with sensitivity to all three DDR inhibitors used in this study. However, our data suggest that different BER or SSBR genes may show distinct genetic interactions with these three inhibitors. These results raise an interesting question: how defects in each step of BER repair lead to the sensitization to different DDR inhibitors? Additionally, using inhibitors targeting different activities of APEX1, we found that both the REDOX activity and endonuclease activity of APEX1 are involved in DNAPKi sensitivity. Additional experiments are needed to further elucidate the mechanisms underlying this sensitization.

Several DDR-inhibitor combinations, such as ATRi plus PARPi, are being proposed and/or tested in the clinic for the treatment of breast and ovarian cancers. In this study, we compared different combinations and showed that the ATMi plus PARPi combination has the highest synergistic effect. Additionally, we conducted CRISPR screens and found that this synergy is likely due to increased apoptosis caused by the dramatic accumulation of DNA damage. We reason that this combination should be tested in the clinic, especially for breast, ovarian, pancreatic, and prostate cancers, in which PARPi-based therapies have already been approved and/or shown to be effective.

Notably, our work provides a global view of genetic interactions with DDR inhibitors in only one cell line. Nevertheless this approach allows comparisons among different DDR inhibitors. It is likely that cells from different genetic backgrounds, such as cells from different tissue origins or cancer cells with specific mutations, may display distinct patterns of genetic interactions with these DDR inhibitors. Furthermore, although we cannot exclude potential off-target effects of these inhibitors, we included mTORi as a control. We expect that gene–drug interaction studies will include similar or other controls to eliminate or at least reduce potential off-target effects of any particular inhibitors.

In sum, the results from this study allow us to compare genetic vulnerabilities to different DDR inhibitors. These data suggest possible biomarkers (e.g. *YWHAE* loss for CHK1i therapy) or possible combination therapies (e.g. ATMi plus PARPi) that warrant further validation. Our data also indicate complex relationships between and within known DNA repair pathways that may be difficult to characterize through the study of individual genes (e.g. possible competition between the BRCA1-A and BRCA1-CtIP complexes in HR repair and the distinct roles of each nucleotide excision repair/SSBR gene in cell survival following treatment with different DDR inhibitors). Furthermore, this approach can be extended to include other inhibitors targeting the same genes/kinases and/or cells with different genetic backgrounds to further enhance our understanding of DDR pathways and different inhibitors targeting these pathways.

## Supplementary Material

gkab643_Supplemental_Files
