# Supplementary material for: Genetic vulnerabilities upon inhibition of DNA damage response
Source: Nucleic Acids Res. 2021 Jul 28;49(14):8214–31. doi: 10.1093/nar/gkab643 (PMC8373146; doi:10.1093/nar/gkab643)
Supplement: gkab643_Supplemental_Files [file gkab643_supplemental_files.zip › Supplementary Figure Legends_CW07062021.docx]

**SUPPLEMENTARY INFORMATION**

**Supplementary Figure 1: (Related to Figure 1)**

1. Inhibitors used in the CRISPR screens and their working concentrations.
2. Validation of the efficacy of these inhibitors at IC_20_ concentrations. HEK293A cells were pretreated with different inhibitors at the indicated concentrations for 24 hrs and then were treated with HU (8 mM, 3 hrs), CPT (200 nM, 3 hrs), or IR (6 Gy, 3 hrs). Cell lysates were collected and Western blotting was conducted with the indicated antibodies. Abbreviations: HU, hydroxyurea; CPT, camptothecin; IR, ionizing radiation.
3. HEK293A cells were pretreated with different inhibitors at the indicated concentrations. At the indicated time points, cells were collected, fixed with ethanol, and stained with propidium iodide. Fluorescence-activated cell sorting analyses were then performed.

**Supplementary Figure 2: (Related to Figure 1)**

1. Comparison of different CRISPR screen results. The inhibitors and cell lines used were presented. The criteria for the hit selection were listed.
2. Synthetic lethal (sensitive) genes and synthetic survival (resistant) genes identified from the four ATMi screen datasets were compared with the use of Venn diagram. The genes identified from three or more screens were listed. The genes identified by all the screens were marked by red dashed circle. The enriched complexes were marked by black dashed circle.
3. Synthetic lethal (sensitive) genes and synthetic survival (resistant) genes identified from the four CHK1i screen datasets were compared with the use of Venn diagram. The genes identified from three or more screens were listed. The genes identified by all the screens were marked by red dashed circle. The enrich complexes were marked by black dashed circle.
4. Synthetic lethal (sensitive) genes and synthetic survival (resistant) genes identified from the four ATRi screen datasets were compared with the use of Venn diagram. The genes identified from three or more screens were listed. The genes identified by all the screens were marked by red dashed circle. The enrich complexes were marked by black dashed circle.
5. Curated gene list from the (B)(C)(D). Several common genes shared from different inhibitor screens were listed.

**Supplementary Figure 3: (Related to Figure 2)**

1. Normalized sgRNA fold changes of different 14-3-3 family members in CHK1i group versus DMSO control group from the screens conducted in HEK293A and HCT116 cells. The fold change comes from sgRNA counts in CHK1i-treated group divided by sgRNA counts in DMSO group in the indicated cell lines.
2. Loss of *YWHAE* sensitizes cells to CHK1i treatment as determined by crystal violet cell viability assays. HCT116 cells transfected with control sgRNA or sgRNAs targeting *YWHAE* were exposed to the indicated concentrations of CHK1i inhibitor and grew for 7 days before they were fixed and stained (1500 cells per well in 12-well plates).
3. Clonogenic survival assays with the same cells treated with different concentrations of CHK1i. The mean and s.d. of n = 3 technical replicates are shown.
4. Flow cytometry analysis of cell death of HEK293A WT and YWHAE KO cells under different treatments (DMSO, CHK1i 0.4 µM). Cells were treated as 2 days. Cells were then stained with Annexin-V-FITC and PI, which were further analyzed by FACS. Cells were distinguished into different groups: Q1-Dead cells, Q2-Late Apoptosis cells, Q3-Early apoptosis cell, Q4-Living cells. Representative results were shown in the left panel. Quantification of the experiments were shown in the right panel. The mean and s.d. of n = 3 independent experiments are shown, n=3. ****P* < 0.001, ***P* < 0.01, n.s.=no significant change, student t-test.
5. The quantification of cell cycle phases in each group. The cell cycle phases were calculated with Watson model in Flowjo. Different cell cycle phases were marked in the indicated colors.
6. 293A WT and *YWHAE* KO were treated with or without CHK1i (0.8 µM) for 24 hrs. Then the cells were incubated with CIdU (25 µM) for 20 mins and then treated with IdU (250 µM) for 20 mins. DNA fibers from these cells are indicated. The scale bar represents 10 μM.
7. The quantification of DNA fiber lengths. Both CIdU and IdU fibers were counted, 200 fibers per group were counted. The mean and s.d. of are shown, n.s. = not significant, ****P* < 0.001, Student *t*-test. The data was representative of two independent experiments.
8. Representative dose-response survival curves of HEK293A wild type (WT) or YWHAE-knockout (KO) cells exposed to increasing concentrations of CHK1i or CHK1i+CDK2i (CVT313 2µM). The mean and s.d. of n = 3 technical replicates are shown. The IC_50_ value of CHK1i in each group is shown on the right side of the panel. (The mean and s.d. of n = 3 independent experiments are shown). ***P* < 0.01; Student *t*-test.
9. Loss of *YWHAE* sensitizes cells to ATRi treatment as determined by crystal violet cell viability assays. HEK293A WT cells or YWHAEKO cells were exposed to the indicated concentrations of ATRi inhibitor and grew for 14 days before they were fixed and stained (200 cells per well in 6-well plates).

**Supplementary Figure 4: (Related to Figure 3)**

1. Positions of sgRNAs used in this study and the generation and validation of HEK293A-*KLHL15* KO cells by sequencing using sgRNA2.
2. Western blotting results of the indicated samples.
3. Crystal violet viability assays of the indicated cells. HEK293A cells were transfected with the indicated constructs and/or siRNAs and then exposed to the indicated ATMi treatment for 7 days (1500 cells per well in 12-well plates).
4. Clonogenic survival assays with the same cells treated with different concentrations of ATMi. The mean and s.d. of n = 3 technical replicates are shown.
5. U2OS-DR-GFP cells were infected with lentivirus-encoding control sgRNA or sgRNA targeting *KLHL15* together with/without *CtIP* siRNA. The cells were then transfected with I-SceI; 48 hrs after transfection, cells were harvested and assayed for GFP expression using FACS. Representative data from one experiment are shown. The GFP-positive cells were gated. Quantification of these experiments is shown in the middle of this panel. The mean and s.d. of n = 3 independent experiments are shown; ***P* < 0.01; ****P* < 0.001; Student *t*-test. The protein levels of each gene were detected with Western blotting and shown on the right side of the panel.
6. Crystal violet viability assays of the indicated cells. The indicated cells were exposed to the indicated ATMi treatment for 14 days (200 cells per well in 6-well plates). The protein levels of the indicated genes were detected by Western blotting and shown on the right side of the panel.

**Supplementary Figure 5: (Related to Figure 4)**

1. Dose-response survival curves of HEK293A wild type (WT) or ATM knock-down cells exposed to increasing concentrations of ATMi (AZD1390). The mean and s.d. of n = 3 technical replicates are shown.
2. Dose-response survival curves of HEK293A wild type (WT) or ATM-knockout (KO) cells exposed to increasing concentrations of PARPi (Olaparib) or Olaparib+ATMi (AZD0156 0.05µM). The mean and s.d. of n = 3 technical replicates are shown.
3. Dose-response survival curves of HEK293A cells exposed to increasing concentrations of PARPi (Olaparib) or Olaparib+ATMi (AZD1390 0.2µM). The mean and s.d. of n = 3 technical replicates are shown.
4. Dose-response survival curves of HEK293A wild type (WT) or ATM knock-down cells exposed to increasing concentrations of ATMi (AZD0156). The mean and s.d. of n = 3 technical replicates are shown. The protein levels of indicated genes were detected with Western blotting and shown on the right side of the panel.

**Supplementary Table 1: Drug-Z scores of the CRIPSR screens with different inhibitors.**

**Supplementary Table 2: Comparison between different CRIPSR screen datasets.**

**Supplementary Table 3: Profiles of proteins in HEK293A WT cells and *KLHL15* KO cells.**
