# Supplementary material for: Genetic vulnerabilities upon inhibition of DNA damage response
Source: Nucleic Acids Res. 2021 Jul 28;49(14):8214–31. doi: 10.1093/nar/gkab643 (PMC8373146; doi:10.1093/nar/gkab643)

Supplementary Figure 1: related to Figure 1

A

Target	Agents	Concentration Used in Screens
ATR	AZD6738	0.4uM
ATM	AZD0156	0.05uM, 0.2uM
DNAPK	NU7441	3uM
mTOR	Rapamycin	50nM
CHK1	LY2603618	0.4uM

B

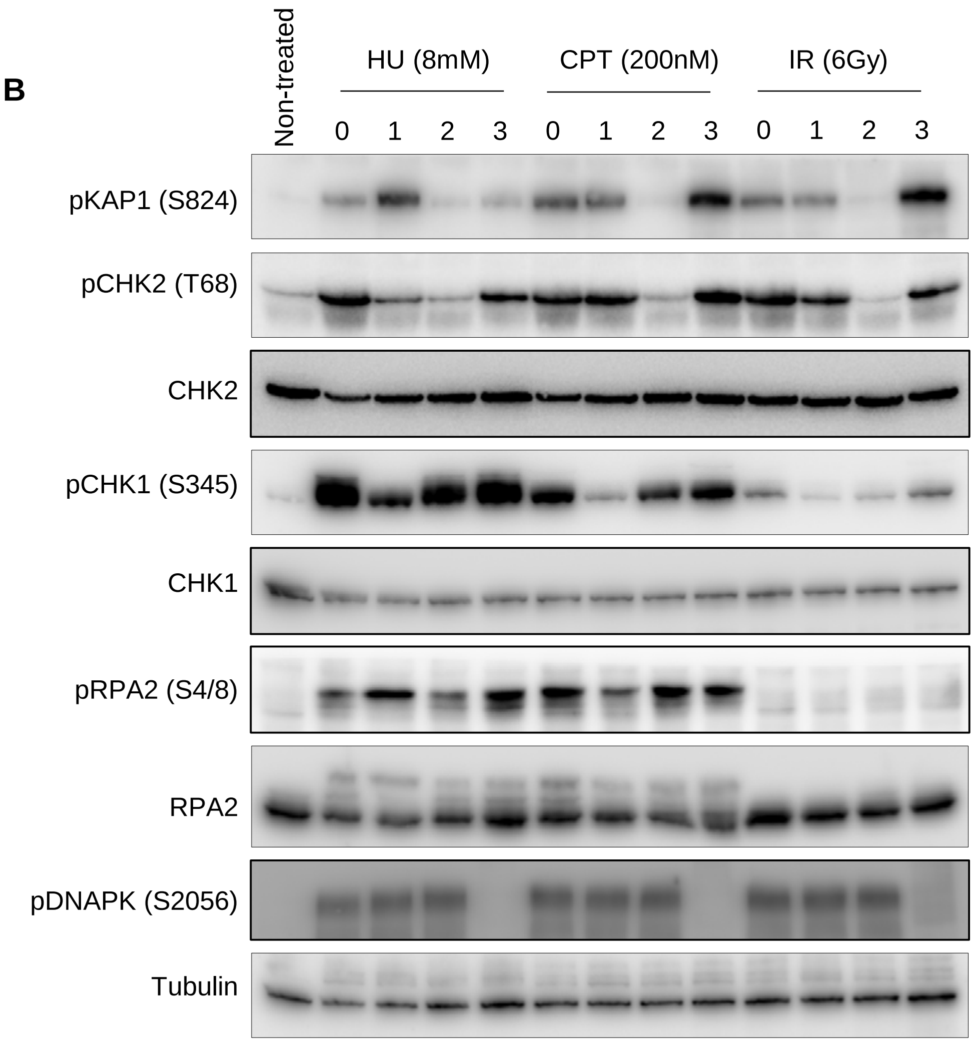

0: DMSO
1: ATRi (AZD6738, 0.4μM, **IC20**)
2: ATMi (AZD0156, 0.05μM, **IC20**)
3: DNAPKi (NU7441, 3μM, **IC20**)

C

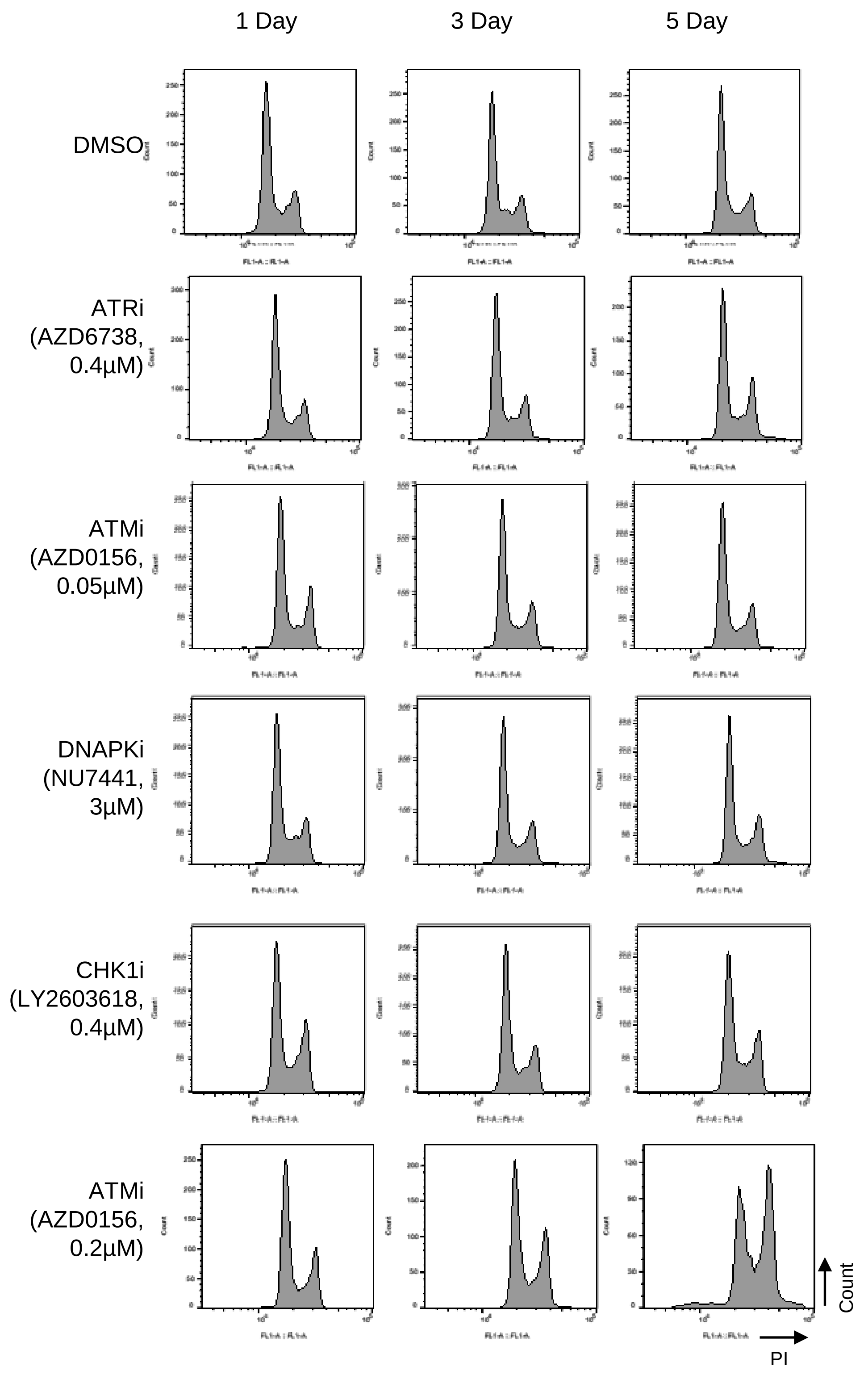

Supplementary Figure 2: related to Figure 1

A

Target	Inhibitors	Cell Line	Library	Reference	Hits Criteria
ATM	M3541	A549; H460	Brunello v2	Cai M.Y. et al, 2020	For results from Brunello v2 library, the synthetic lethal hits and synthetic survival hits were as the same as reported in these studies. For results from TKO v3 library, genes with DrugZ score < -2.5 were marked as synthetic lethal (sensitive) hits; genes with DrugZ score >2.5 were marked as synthetic survival (resistant) hits.
ATR	VE821; AZD6738	HeLa; HCT116; RPE-hTERT TP53-/-	TKOv3	Hustedt N. et al, 2019	
ATR	AZD6738	HEK293A; HCT116; MCF10A	TKOv3	Wang C. et al, 2019	
CHK1	Prexasertib	A549; H460	Brunello v2	Li F. et al, 2020	
ATM	AZD0156	HEK293A	TKOv3	This Study	
CHK1	LY2603618	HEK293A; HCT116	TKOv3	This Study	
DNAPK	NU7441	HEK293A	TKOv3	This Study	

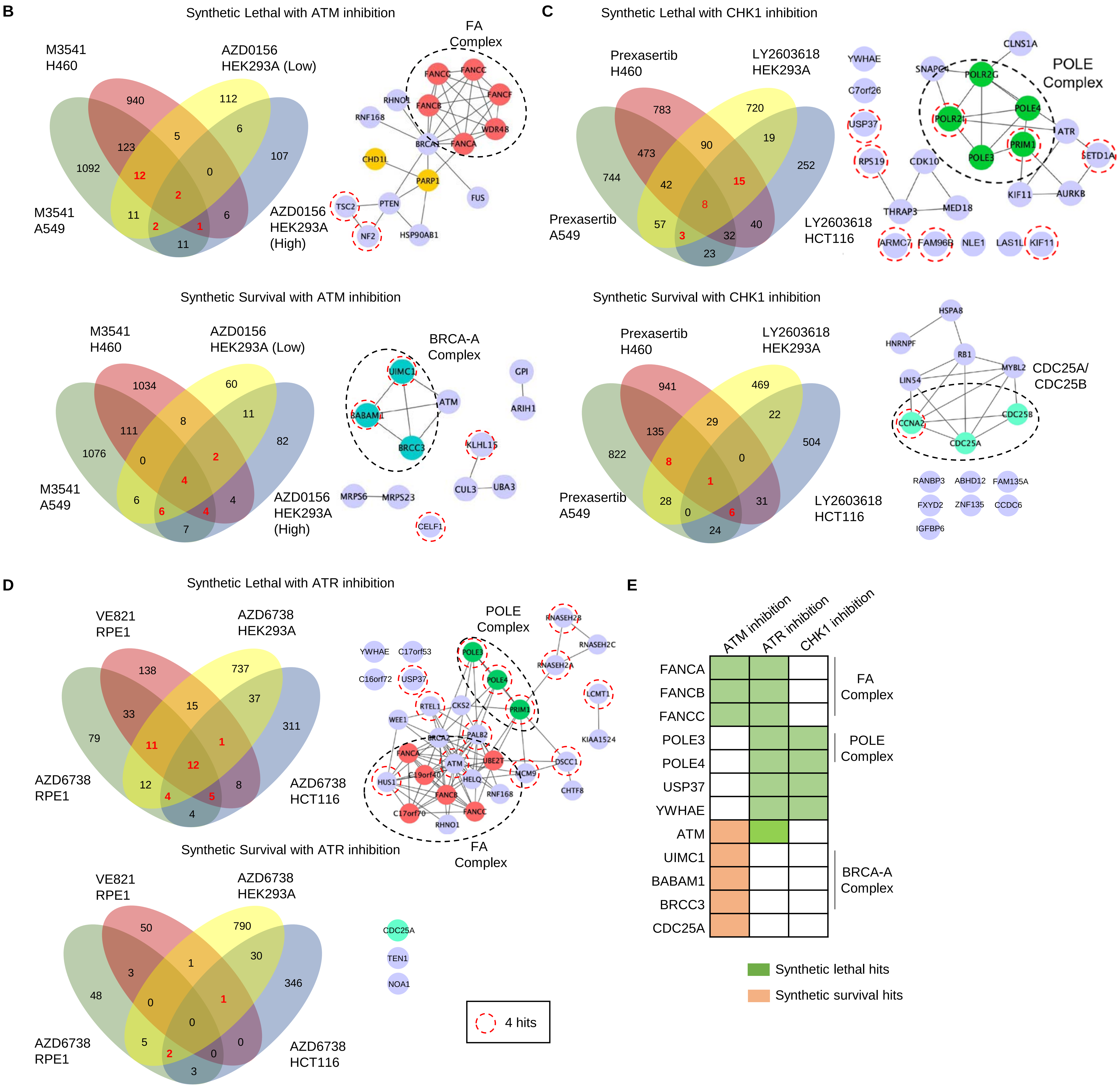

Supplementary Figure3 : Related to Figure 2

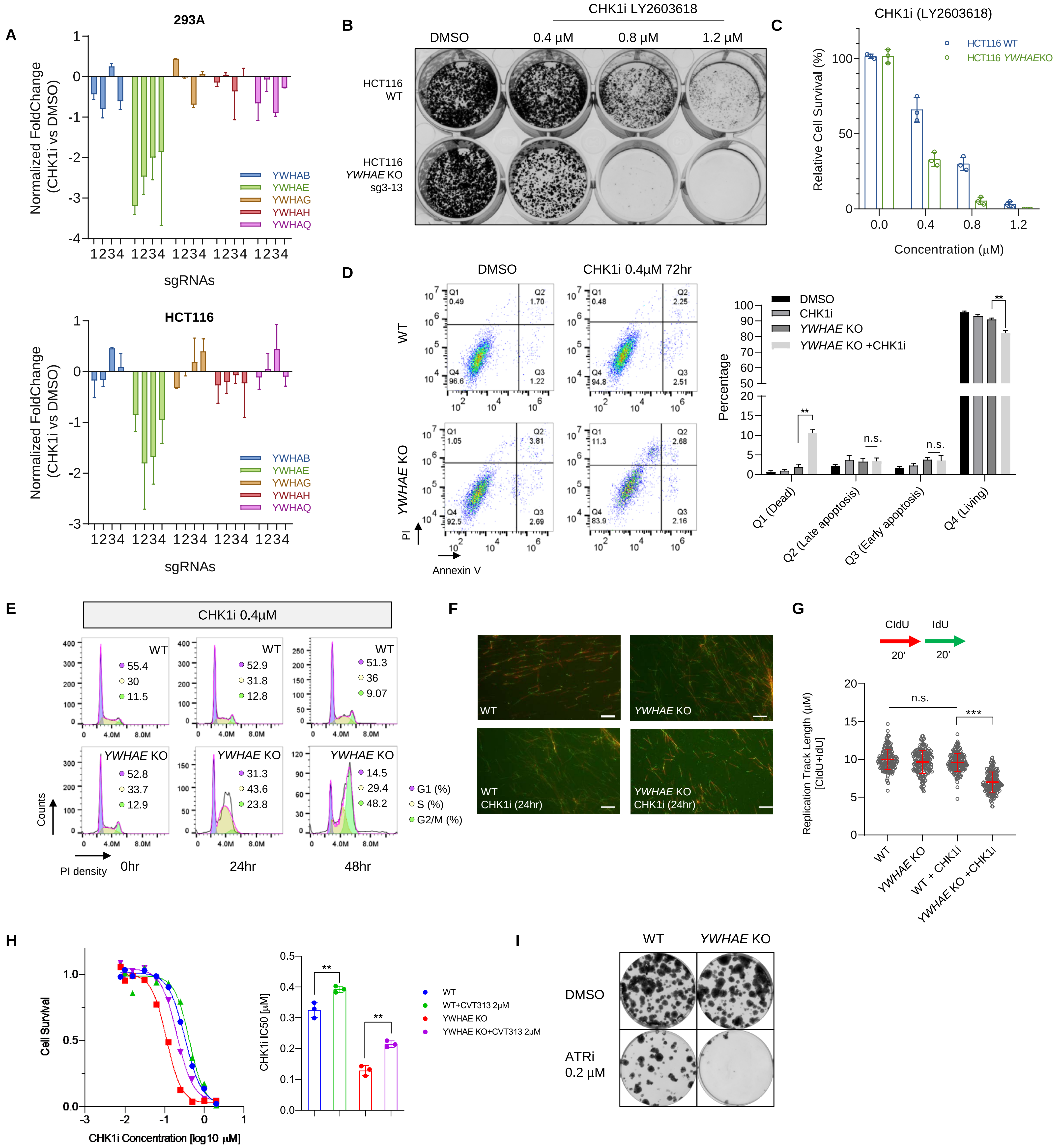

Supplementary Figure 4: related to Figure 3

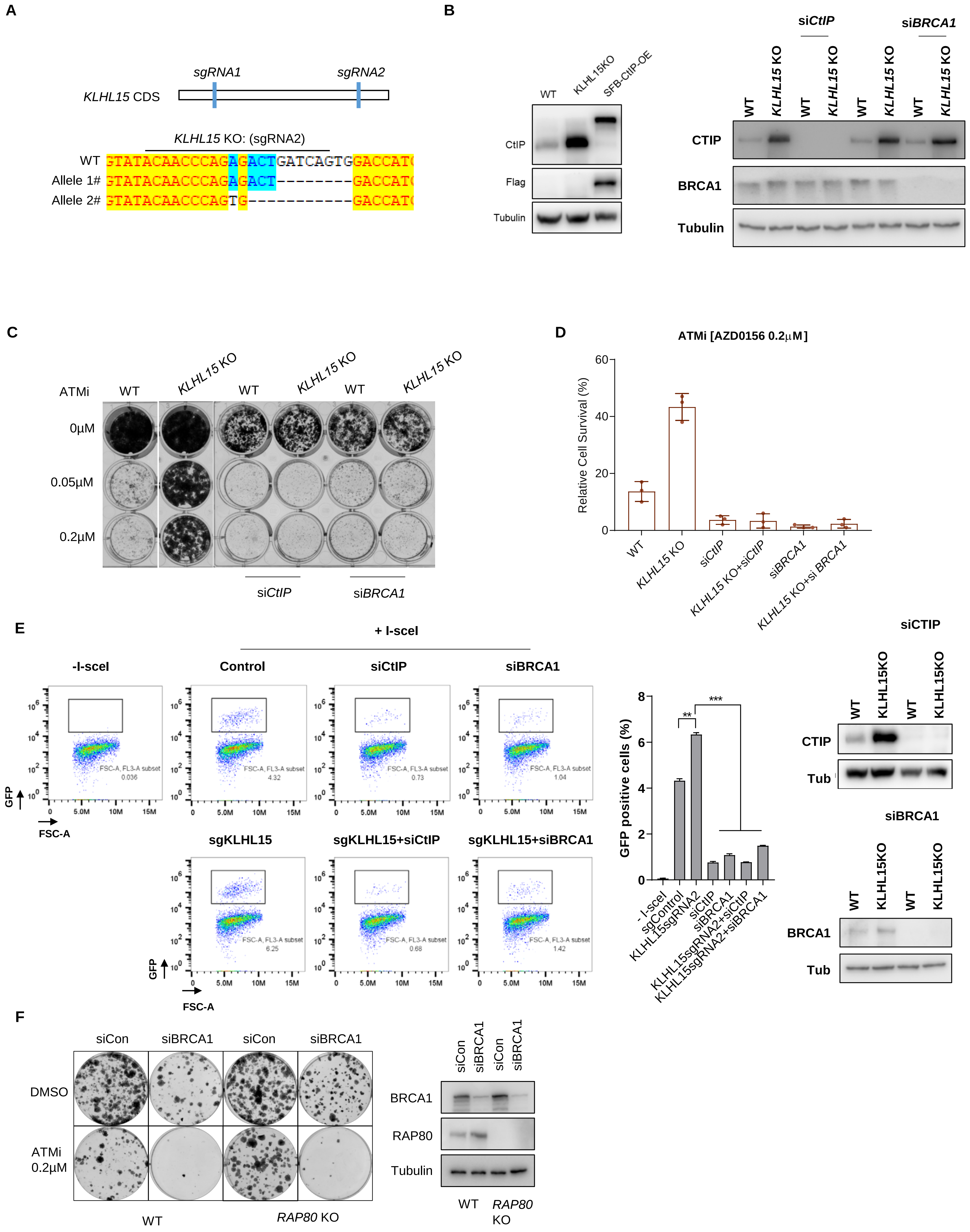

Supplementary Figure 5: related to Figure 4

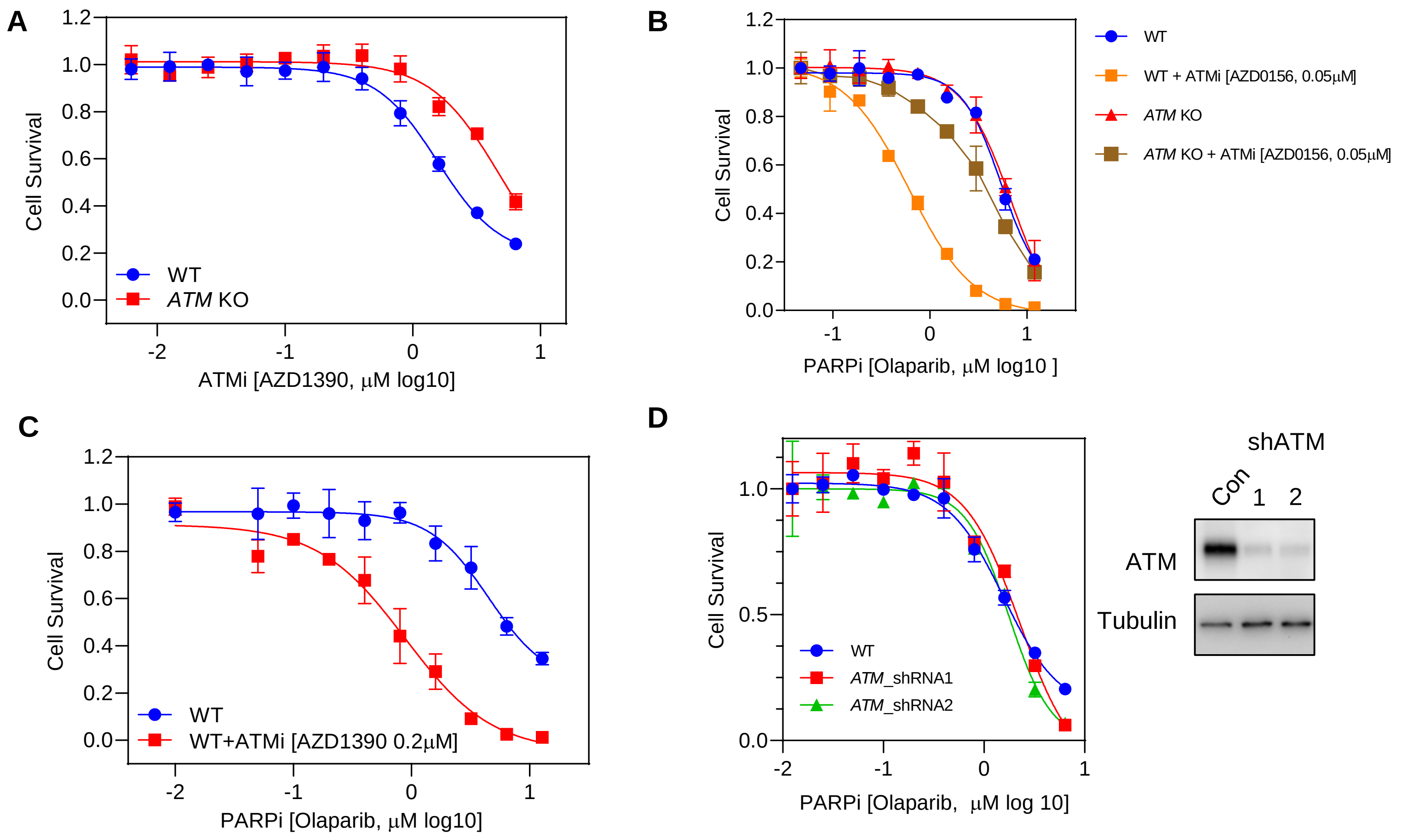

Supplement: gkab643_Supplemental_Files [file gkab643_supplemental_files.zip › Supplementary Figures.pdf]
